# Future health technology trends, policy, and governance perspective: the Turkish case

**DOI:** 10.1186/s12961-024-01217-4

**Published:** 2024-10-29

**Authors:** Elif Sena Kambur, Hasan Hüseyin Yıldırım

**Affiliations:** Gülhane Faculty of Health Sciences, Department of Health Management, University of Health Sciences, Ankara, Türkiye

**Keywords:** Health, Health policy, Health systems, Health ecosystem, Health technology

## Abstract

**Background:**

Advanced health technologies that emerge with the development of technology have an impact on health systems. This study aimed to determine the effects of these technologies on Türkiye’s health system and present policy recommendations to reshape Türkiye’s health system and policies accordingly.

**Methods:**

Interviews were conducted with senior managers, bureaucrats, policy-makers and decision-makers from seven different institutions on the subject. Content analysis was performed on the data obtained and evaluative categories were established.

**Results:**

It was concluded that these technologies would not have a positive impact on two identified themes, a negative impact on seven themes and a predominant impact on five themes in Türkiye.

**Conclusions:**

To adapt to the new health ecosystem in Türkiye, it is recommended to increase digital literacy, conduct economic evaluations of technologies, promote domestic production, ensure up-to-date follow-up, collaborate with the engineering field, enhance health technology evaluation practices, improve access to technologies and ensure that the infrastructures of health institutions are compatible with technologies. Various policy suggestions have been presented for the development of Türkiye’s health system.

## Highlights

- We believe that the healthcare ecosystem of the future will revolve around three main elements.
- Challenges in health, health technologies and health policies.
- Our focus is on understanding how future health technologies will impact health systems.
- We provide policy recommendations to address these impacts.

## Introduction

Improving the health levels of societies is achieved through health systems [1]. Health systems, along with all their subsystems, constitute the health sector. The health sector is a dynamic sector that is influenced by various changes and transformations occurring in the world [2, 3]. The methods, techniques and equipment used within the healthcare system change and develop, influenced by these changes. Practices such as herbalism and healing, which were prevalent in the early periods of humanity, have now been replaced by advanced health technologies. With the rapid progress in technology, the health ecosystem has evolved significantly compared with previous centuries [4, 5].

It is assumed that the new health ecosystem will be shaped around three elements [6]. The first factor is the challenges experienced in the field of health on a global scale. These challenges include the ageing of the population; the increasing burden of chronic diseases; changing needs, demands, desires and expectations; rising health expenditures; epidemics; and technological developments [6–10]. Another element of the ecosystem is the technology element, which presents both challenges and solutions in the field of health [10–13]. Advanced health technologies, which are seen as one of the trends that will shape the new health ecosystem are discussed in three groups: biotechnology, digital health and innovative machines. Technologies falling under the biotechnology category include gene/genome/gene therapy, nanomedicine, vaccines, personalized medicine, stem cell medicine, biobank, bioinformatics and insulins. Digital health technologies encompass big data, the internet of things, blockchain, telemedicine, remote patient monitoring systems, virtual and augmented reality, open technology ecosystems, artificial intelligence, 5G, cloud technology, wearable technologies, smartphone applications, sensors, interventional and rehabilitation robotics and electronic health records. Innovative machines include drones, unmanned aerial vehicles, machine learning, 3D printers, robots, driverless cars and mobile devices [14]. It is believed that the current traditional structure of the health sector is unable to adequately address the challenges it faces. Therefore, it is argued that the primary driving force behind advancements in health technologies is to overcome these challenges. [15]. According to the literature, there are suggestions that health technologies should be utilized in health policy-making to address the challenges present in the field of health [16–18]. As a result, it is proposed that the third element that will shape the future health ecosystem is policy and governance. Subelements that make up policy and governance include access, financing, service delivery, regulation, manpower, ethics, equity and social responsibilities, biosecurity, cybersecurity, local, domestic and national production in the health industry, digital health literacy and management of expectations [14].

For the future development of the health ecosystem, countries around the world have begun to undergo a transformation their health ecosystems. Türkiye took significant steps in adopting these technologies, starting in 2002 [19]. It is crucial for countries to redesign their health systems to align with the future health ecosystem to stay current with advancements and effectively meet changing needs and demands. This study aims to explore the impact of future health technologies on Türkiye’s health system and offer policy recommendations for restructuring Türkiye’s healthcare systems and policies to align with the future health ecosystem.

## Materials and methods

The aim of the study is to determine the effects of advanced health technologies, which are believed to shape the future health ecosystem, on Türkiye’s health system. The goal is to offer policy recommendations for redesigning Türkiye’s health system and policies to align with the future health ecosystem. When reviewing the literature, it is evident that there are studies on the challenges faced by the health sector (see [20, 21]), advanced health technologies used in the health sector (see [22, 23]) and health policies (see [24, 25]). However, there appears to be a lack of studies on the effects of these technologies, proposed as solutions to the challenges in the health sector, on Türkiye’s health system and in the redesign of its health system and policies in relation to these technologies. Therefore, the study is considered important in terms of contributing to the existing literature.

The research was planned as a qualitative study, using the criterion sampling method, one of the purposeful sampling methods. Seven participants, including senior managers/bureaucrats, policy-makers and academics working in the fields of health technologies and health policies, were interviewed from seven different institutions including the presidency, Ministry of Health, higher education institutions, universities and the health sector. Data collection took place between 27 March 2023 and 31 June 2023.

**Fig. 1:**
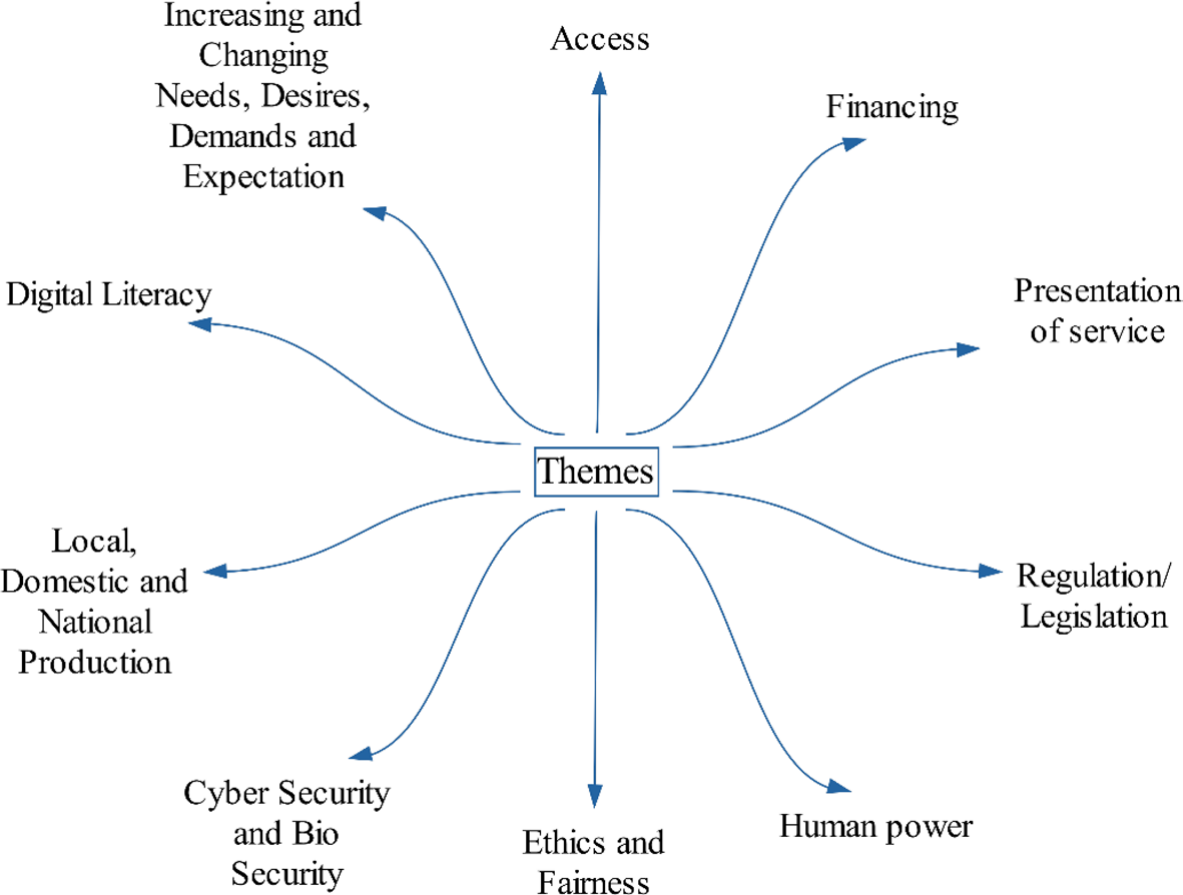
Main themes of research questions

Data were collected from participants from through interviews and were written down. Since the study was conducted in Türkiye, the interview form was prepared in Turkish, and the interviews were conducted in Turkish. The interview form consisted of 13 questions related to health technologies and health policy. Each question focused on a specific health policy element (Fig. 1). The questions were divided into two categories: A and B. Category A questions inquired about the effects of technologies on health policy elements, while Category B questions asked for policy suggestions. The twelfth question addressed any additional issues regarding the future health ecosystem, and the thirteenth question allowed for any additional statements. Before presenting the interview form to participants, it was reviewed by a professor, an associate professor and a doctor faculty member and minor corrections were made to ensure clarity.

Content analysis was conducted using the MAXQDA software program to analyse the data. To ensure reliability, the first author coded the data twice at different times, with any discrepancies being resolved. The second author then reviewed and confirmed the coding. Evaluative categories were developed from this coding process. Themes were identified on the basis of a combination of deductive and inductive analysis methods, as predetermined through a literature review.

## Findings and discussion

### Answers given within the scope of impact category (A) and policy recommendation category (B)

The answers provided by participants within the impact category were divided into three categories: “positive, negative and doubtful/undecided”. Doubtful/undecided responses included sentences with expressions such as “maybe” or “can be expected". The answers within the policy category were also divided into two categories: “increasing–decreasing regulations” if the aim was to increase or decrease an existing practice, and “new regulations” if it was a completely new proposal.

### Access theme

**Fig. 2:**
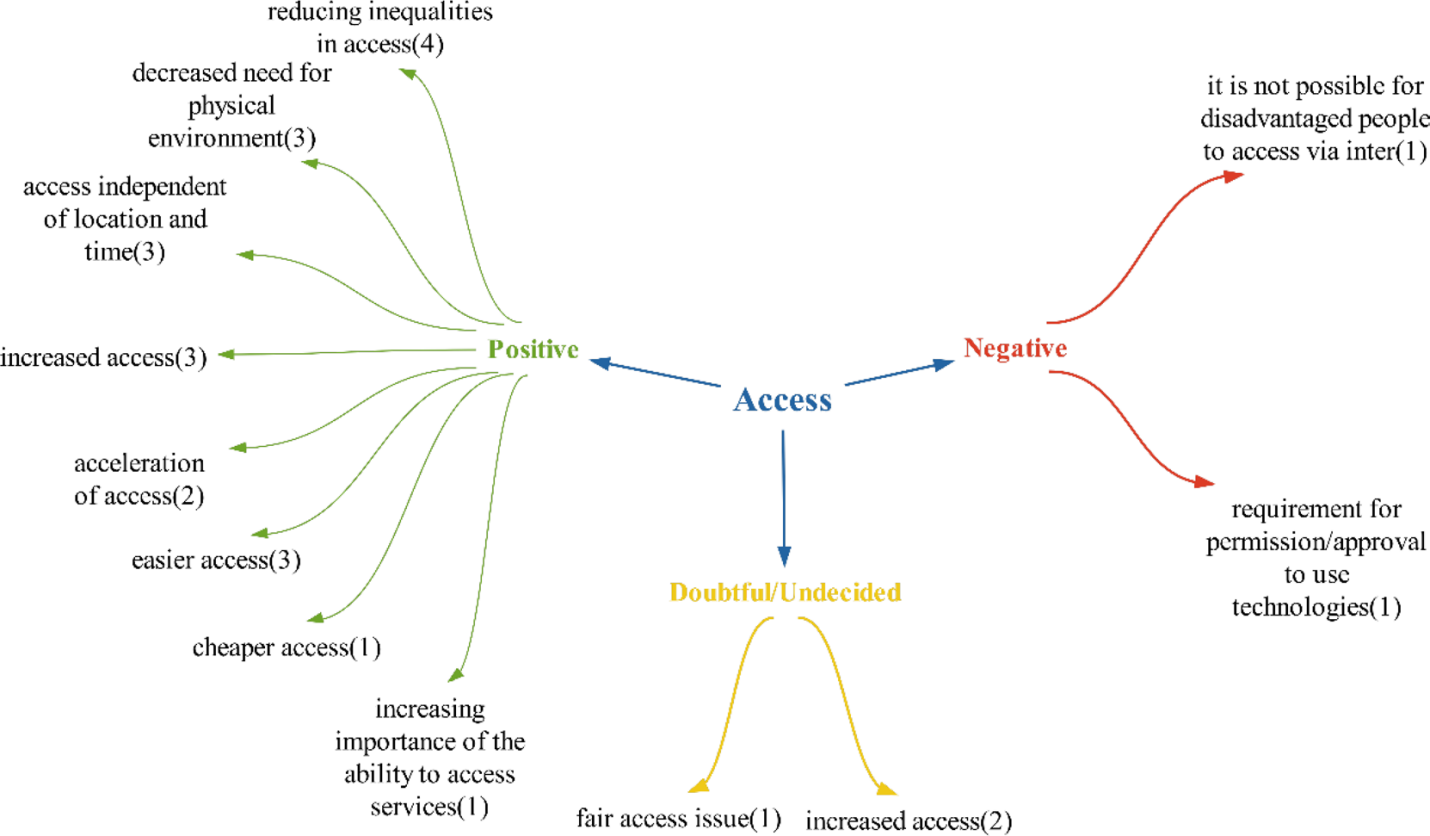
Views on what kind of effects technologies will have on access to health services

Within the realm of positive effects, Bause et al. discuss the impact of decreasing inequalities in access, while Orhan and Thuemmler and Bai mention the effect of accelerating access. Thuemmler and Bai also highlight the importance of enhancing the ability to access services. Additionally, the studies by Herselman and Yılmaz et al. [11, 26–29]. Emphasize the significance of reducing the need for physical environments. These advancements such as cheaper and increased access to technologies, can diminish the necessity for physical locations, ultimately streamlining access to healthcare services for individuals with hectic schedules by eliminating the time and costs associated with travelling to healthcare facilities (Fig. 2).

However, negative effects related to access include the challenge of reaching disadvantaged populations who may not have internet access, as noted in Sarıhans’s study [30]. Furthermore, the requirement for approval or permission to use certain technologies can hinder access. In response to this issue, the Ministry of Health in Türkiye introduced the Regulation on the Provision of Remote Health Services in 2022. This regulation outlines which health institutions have the necessary infrastructure to offer remote health services and specifies the types of services they can provide [31].

**Fig. 3:**
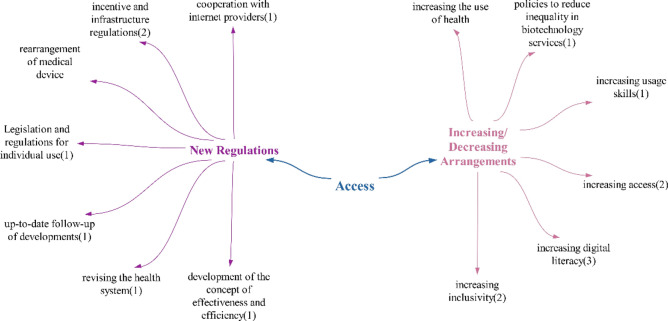
Policy recommendations regarding the impacts of technologies on access

Doubtful/undecided effects are not distinguished from positive or negative effects. Therefore, it was concluded that technologies will mainly have a positive impact on access to health services, given the abundance of positive effects (Fig. 3)

.

It is observed that the number of new regulations and increasing/decreasing regulations in the policy suggestions provided by participants regarding access to health services are similar. The predominant suggestion is that incentives and infrastructure improvements should be included in new regulations, while digital literacy should be increased within the scope of increasing/decreasing regulations. One noteworthy policy recommendation is to collaborate with internet providers. This suggestion is deemed appropriate, as advancements in technology are shifting health processes to the digital realm, making internet usage essential in the field of healthcare.

### Financing theme

**Fig. 4:**
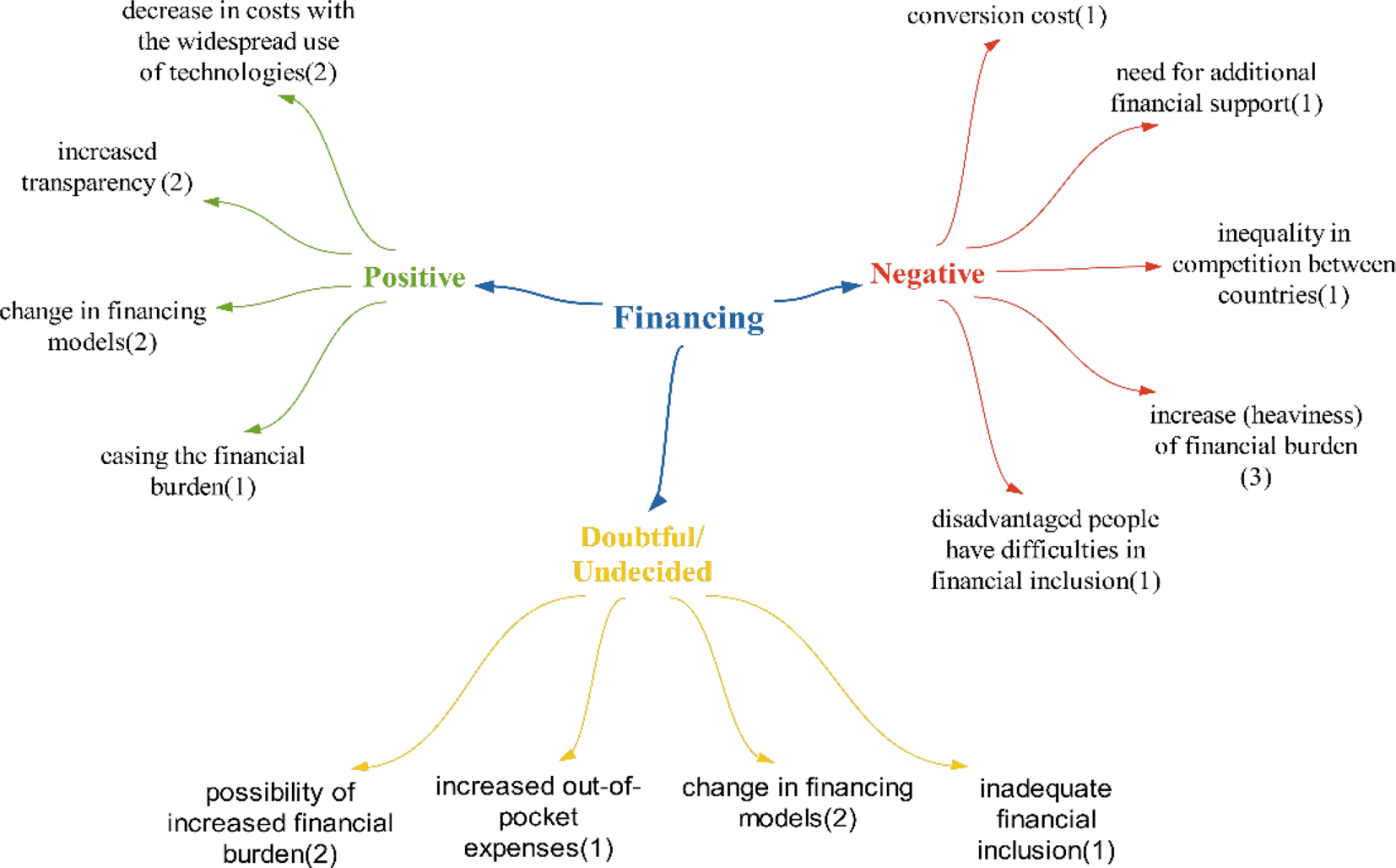
Opinions on what kind of effects technologies will have on the financing of healthcare services

Since the number of positive (seven) and negative (eight) effects in the participants’ statements regarding the impact of technologies on health financing were close to each other, it was concluded that technologies will not have a significant impact on health financing. Within the scope of the positive effects detected in the financing theme, the effect of changes in financing models is similar to the statements in the report published by TUSAP (Türkiye Health Platform) in 2018. The effects of easing the financial burden and reducing costs are similar to the statements in Şimşir and Mete’s study [18, 32]. For the effect of increasing transparency, moving healthcare processes to the digital environment and providing access to information independent of location and time can be expected to increase transparency (Fig. 4)

.

The statement of increased financial burden within the scope of the negative effects detected on finance is similar to the statements in the study of Figueras et al. and the statements in the report published by the Scientific And Technological Research Council of Türkiye (TUBITAK) in 2003. [33, 34] It can be expected that the financial burden will increase, resulting in the “need for additional financial support” as expressed by the participants. The effect of disadvantaged people having difficulty in financial inclusion is that, if financial inclusion cannot be expanded in the face of increasing health expenditures in Türkiye [35], it will be an expected situation that disadvantaged groups will have difficulty in financial inclusion. The effect of inequality in competition between countries is expressed in the studies of Long et al., which states countries need to use technologies to have competitive power [36]. The transformation cost effect refers to the costs that will need to be incurred to transform the new health system in accordance with the technologies.

**Fig. 5:**
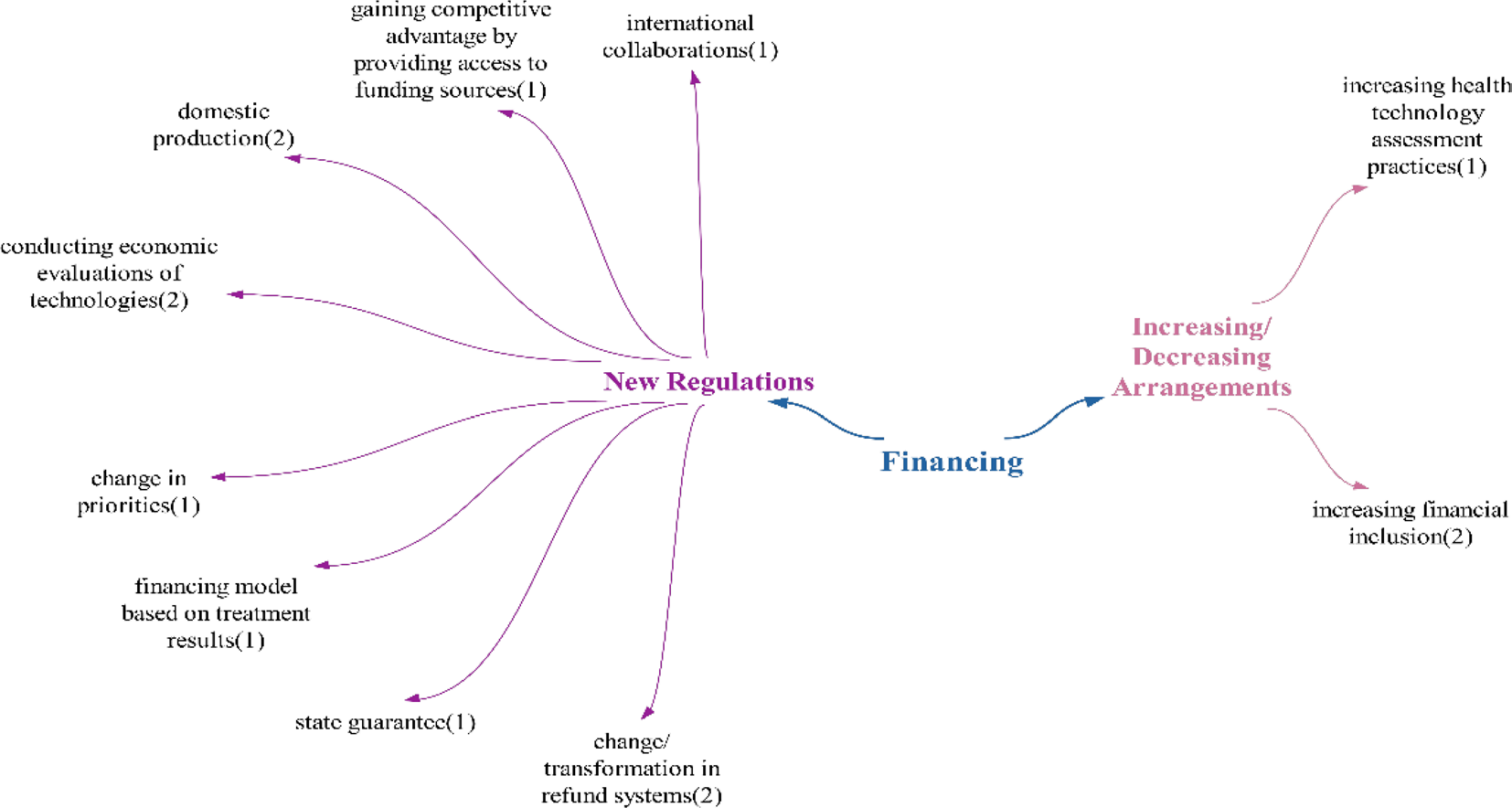
Policy recommendations regarding the effects of technologies on health financing

Within the realm of doubtful/undecided expressions, as opposed to positive and negative effects, out-of-pocket expenses are mentioned. If technologies lead to an increase in financial burden, out-of-pocket expenses are likely to rise. This is because the country may not be able to afford the technologies, and citizens who wish to utilize them will have to bear the costs individually (Fig. 5).

The participants made suggestions regarding the financing of health services, primarily focusing on new regulations. Suggestions included the need for domestic production, economic evaluations of technologies, and changes in reimbursement systems. One proposal aimed at increasing financial inclusion in suggestions for increasing/reducing regulations; one participant suggested that inclusion should be guaranteed not only for disadvantaged groups but also for citizens from other countries:

“…Not only those living within the country, but also citizens living abroad should be included in this scope..”

### Provision of service theme

**Fig. 6:**
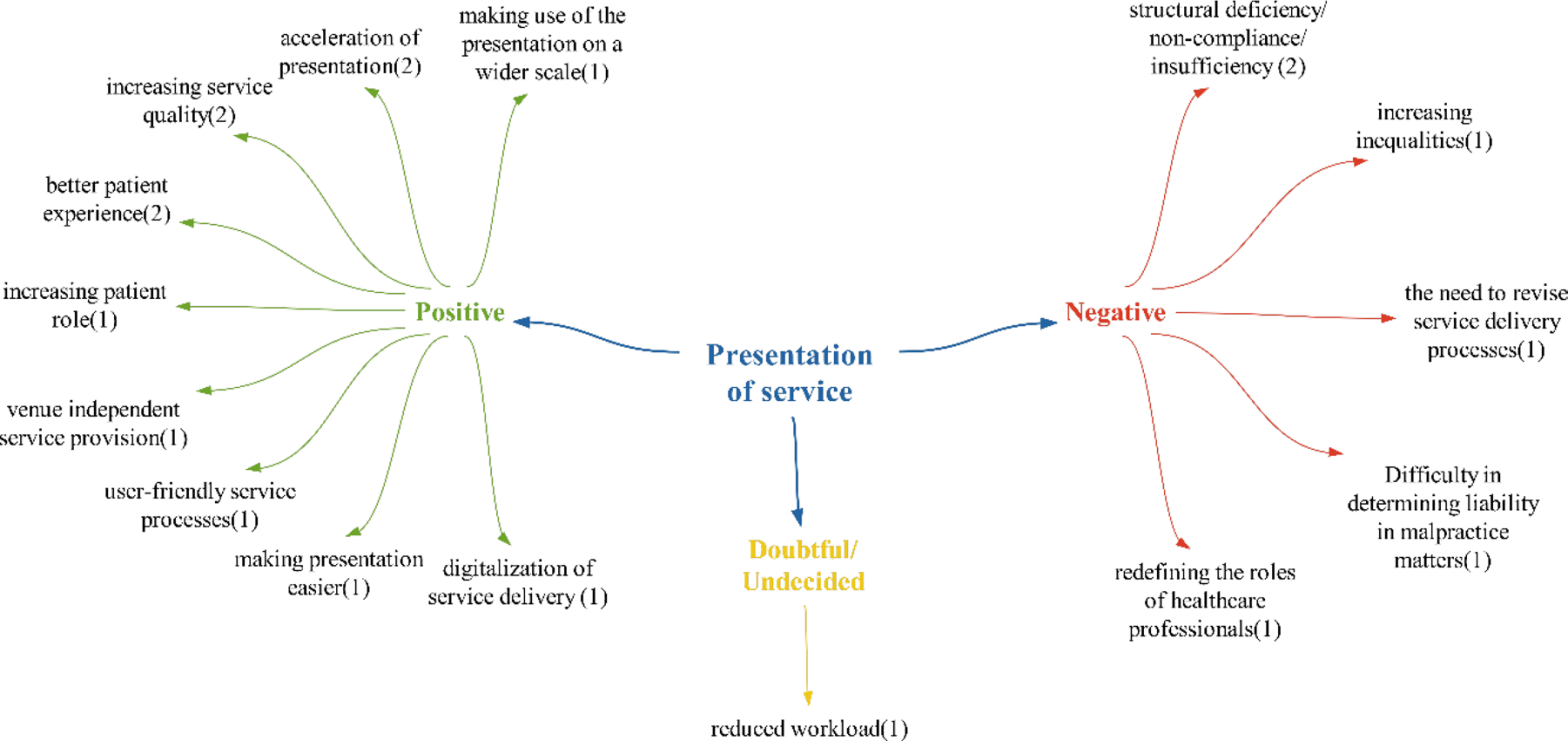
Views on what kind of effects technologies will have on healthcare delivery

In the realm of service delivery, it is anticipated that the positive effects identified will lead to “the benefit of expanding the reach of the presentation” by transitioning the presentation to a digital platform. This shift will allow patients to access service delivery on a broader scale. The “acceleration of the presentation effect” is expected to be facilitated by increased digitalization, which will streamline service delivery by eliminating unnecessary paper/document processes. The enhancement of the quality of the health services provided mirrors findings from Bülbül and Uysal and Ulusinan in existing literature. The growing role of patients aligns with research by Mackay, Bhavnani et al., Bass et al. and Afferni et al. The trend towards location independent service provision is consistent with Kılıç’s study [37–43], [37–43]. The improvements in patient experience, user-friendly service delivery processes and simplified service delivery processes are also in line with the findings of Uysal and Ulusinan. They argue that digitalization simplifies service processes, reduces wait times, replaces paperwork with electronic methods, and fosters interactive communication between patients and their physicians. [42]. Kılıç further suggests that advanced technologies will expedite hospital procedures [41]. The impact of digitalizing of service delivery resonates with the conclusions drawn by Şimşir and Mete [32] (Fig. 6).

The impact of increasing inequalities falls within the realm of negative effects, as indicated in Sarıhan’s study. [30]. The issue of structural deficiency/non-compliance insufficiency of health institutions towards technologies was also addressed in Sargutan’s study [16]. The need to revise service delivery processes is similarly highlighted in the studies of Baem and Kohane, as technologies alter service delivery methods [44]. The effect of making it difficult to determine the responsible person in malpractice cases aligns with the findings of Pasquale and Rowland et al. [45, 46]. The effect of redefining the roles of health professionals was an expected outcome, given that technologies have changed or will change healthcare processes.

**Fig. 7:**
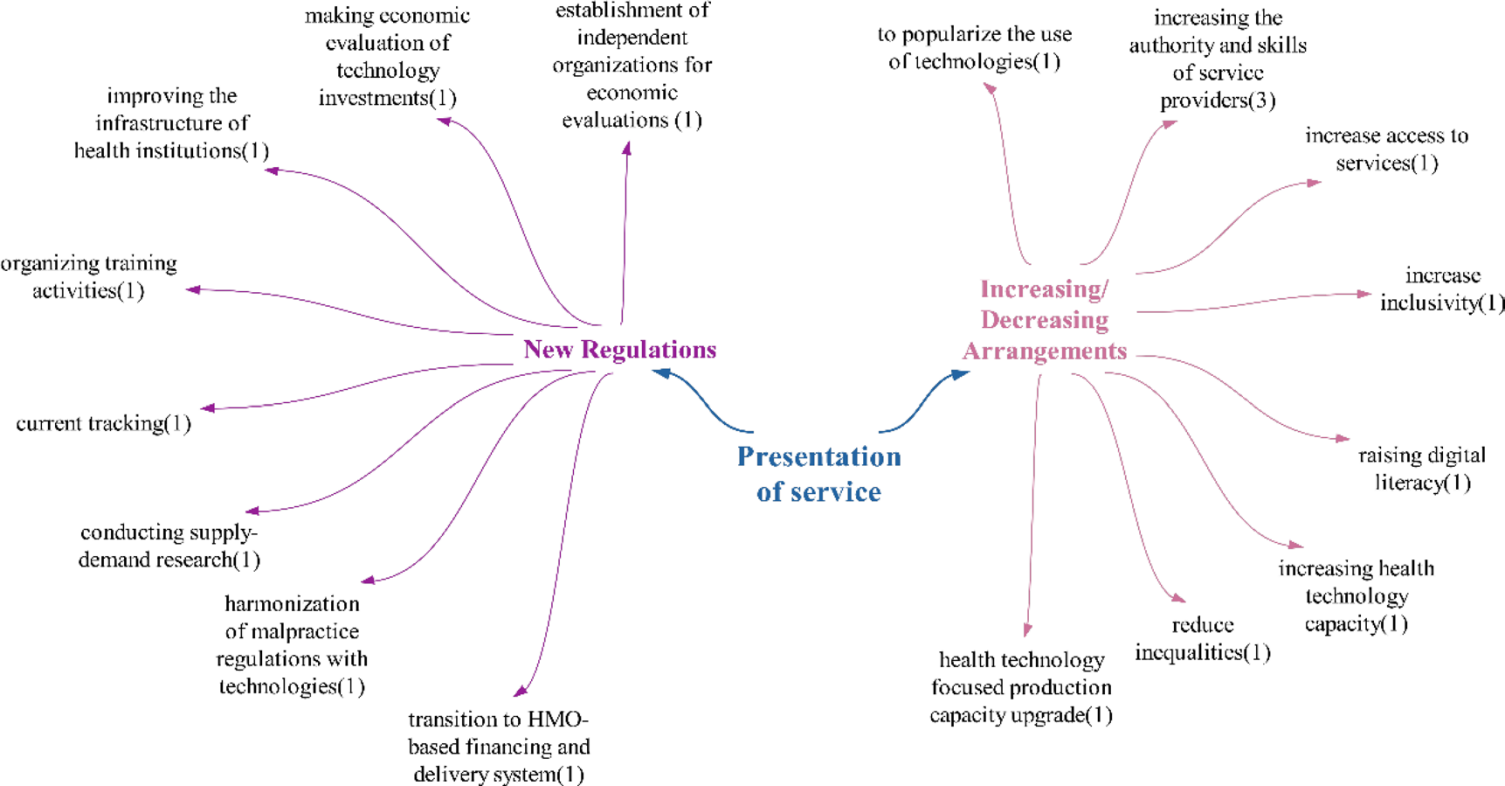
Policy recommendations regarding the impact of technologies on healthcare delivery

Within the scope of doubtful/undetermined effects, only reduction in workload (1) is mentioned. With the ability for healthcare personnel to perform many tasks digitally through digitalization, the effect of reducing workload may be expected. Looking at the statements of the participants, it was concluded that health technologies are likely to have a predominantly positive impact on the provision of healthcare services in the future, as the number of positive effects outweighs the negative ones (Fig. 7).

It is observed that the number of new regulations and increasing/reducing regulations in the participants’ policy suggestions for service delivery are similar. Participants primarily focused on enhancing the authority and skills of service providers within the scope of increasing/reducing regulations. One notable policy recommendation in this area was the shift towards a Health Maintenance Organization (HMO)-based financing and delivery system:

“By transitioning to a Health Maintenance Organization (HMO)-based health financing and delivery system…”

Another significant suggestion within this theme is the creation of independent organizations tasked with conducting economic evaluations of technologies. It is believed that institutions such as National Institute for Health and Care Excellence (NICE) in England could serve as models for these organizations.

Another significant policy proposal in this theme is the harmonization of legal regulations regarding medical malpractice processes with technologies:

“It is recommended that legal regulations concerning medical malpractice align with health technology trends. …”

Given the importance of determining responsibility and sanctions in cases of malpractice in healthcare services involving these technologies, this proposal is also seen as a noteworthy policy recommendation.

### Regulation/legislation theme

**Fig. 8:**
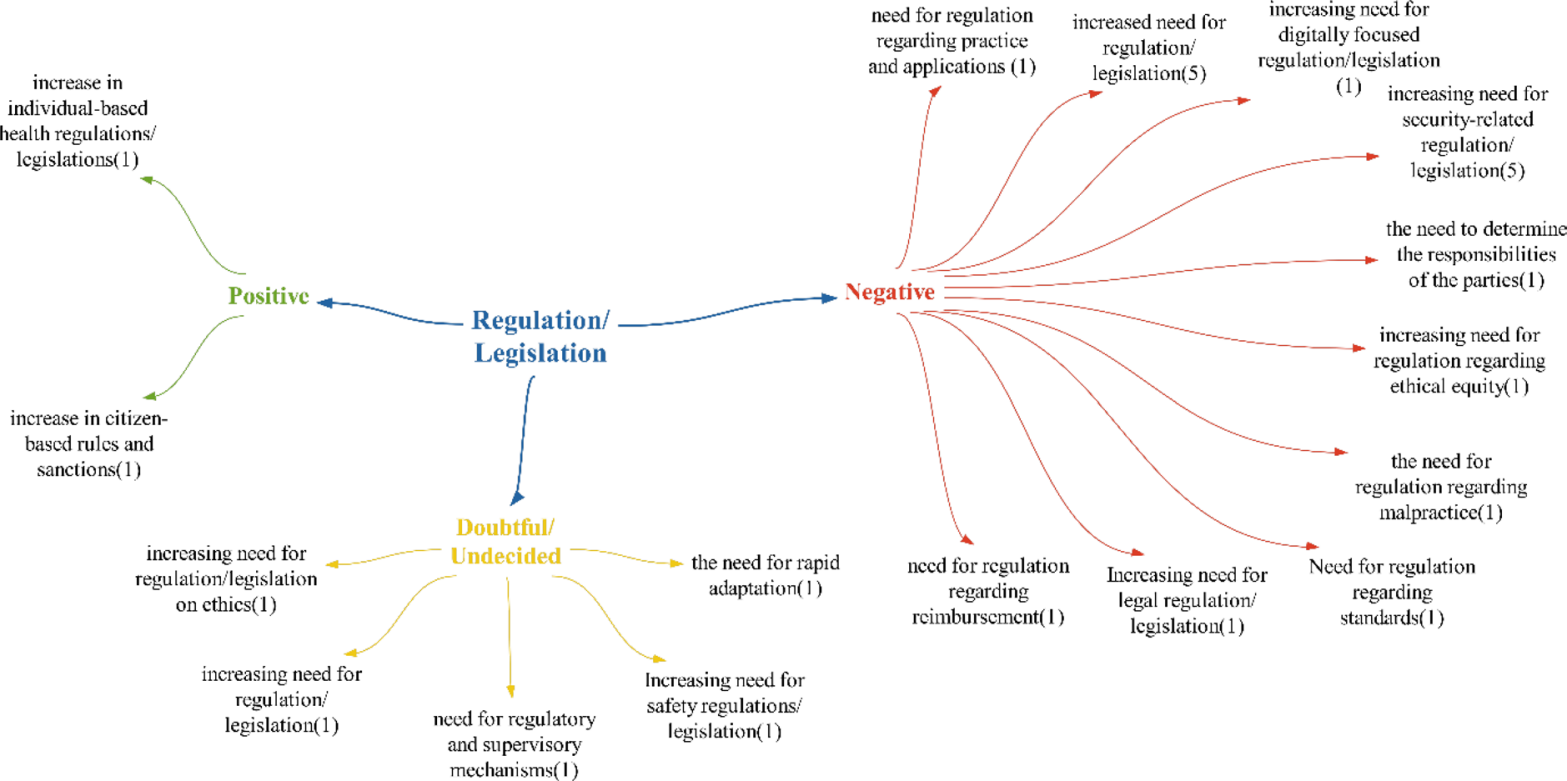
Opinions on what kind of impact technologies will have on health regulations/legislations

When examining the literature in the context of positive effects detected in the theme of regulation, studies by Orhan and Ergenoğlu and Aytuğ state that, similar to developments in the field of health, a patient-centred approach has begun to dominate the health sector [28, 47] (Fig. 8).

The negative effects identified in the regulation theme indicate areas that will require regulation and legislation. It is believed that establishing standards is necessary for the formation of a new system, and digitally focused regulation is needed for health processes to transition to the digital environment. The need for ethical regulations is echoed in studies by Wyatt and Taylor, Hayran, Ay and Özdemir and Bilgin [48–50]. The “need for regulation regarding repayment” is anticipated if changes or additional methods are required on the basis of the results of the financing theme. Legal regulation is deemed essential due to the direct impact of the health field on human life. Regulation concerning practices and applications is crucial for healthcare personnel utilizing new technologies to provide successful healthcare services. Regulation concerning malpractice is vital in determining responsibility in case of potential malpractice. Regarding the “security regulatory need impact”, studies by Baker et al., Kotz et al. and Kreitmair et al. are similar in terms of addressing the issue of security. [51–53]. Similarly, the need for cybersecurity, as highlighted by Kruse et al., Coventry and Branley, and Luh and Yen, underscores the negative impact of technologies on cybersecurity in their studies [54–56].

**Fig. 9:**
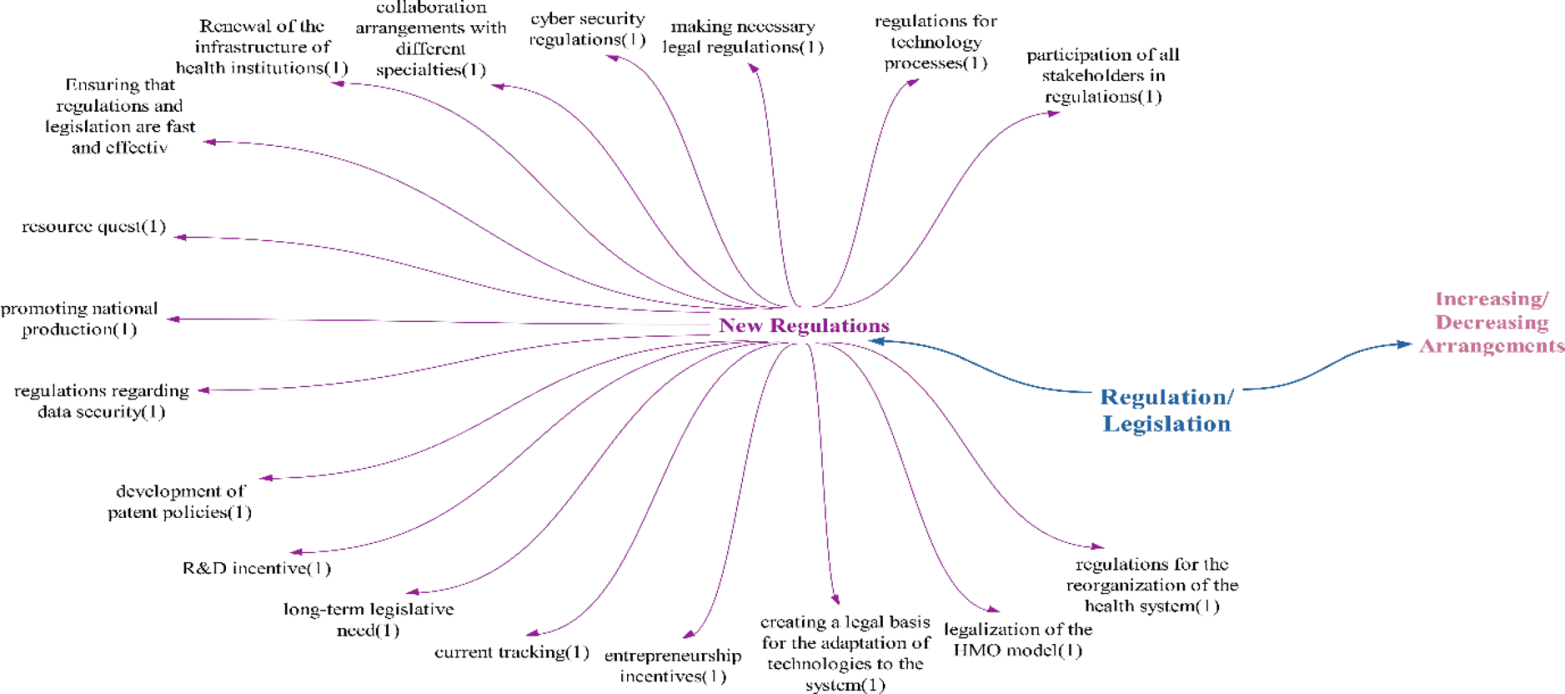
Policy recommendations regarding the impact of technologies on health regulations/legislations

In contrast to the positive and negative effects, the detected suspicious/unstable effects include the need for rapid adaptation and the necessity of regulatory and supervisory mechanisms. Rapid adaptation is seen as a fitting term for the requirement of quickly adaptable regulations to keep up with evolving health technologies. Regulatory and supervisory mechanisms may be needed to oversee the numerous regulations that will be implemented across various areas. On the basis of participant feedback, it was deduced that technologies are likely to have a predominantly negative impact on health regulations/legislations, with a particular focus on security regulations Fig. 9.

Participants provided suggestions for healthcare regulations/legislation solely focusing on new regulations. These recommendations encompass a broad range of policy suggestions, including legal regulations, patent policy development, long-term legislative requirements and entrepreneurship incentives. Given that technologies will impact the entire healthcare system and create a new framework, it was anticipated that regulations in all areas of the healthcare system would need to align with this new structure (Fig. 11).

### Manpower theme

**Fig. 10:**
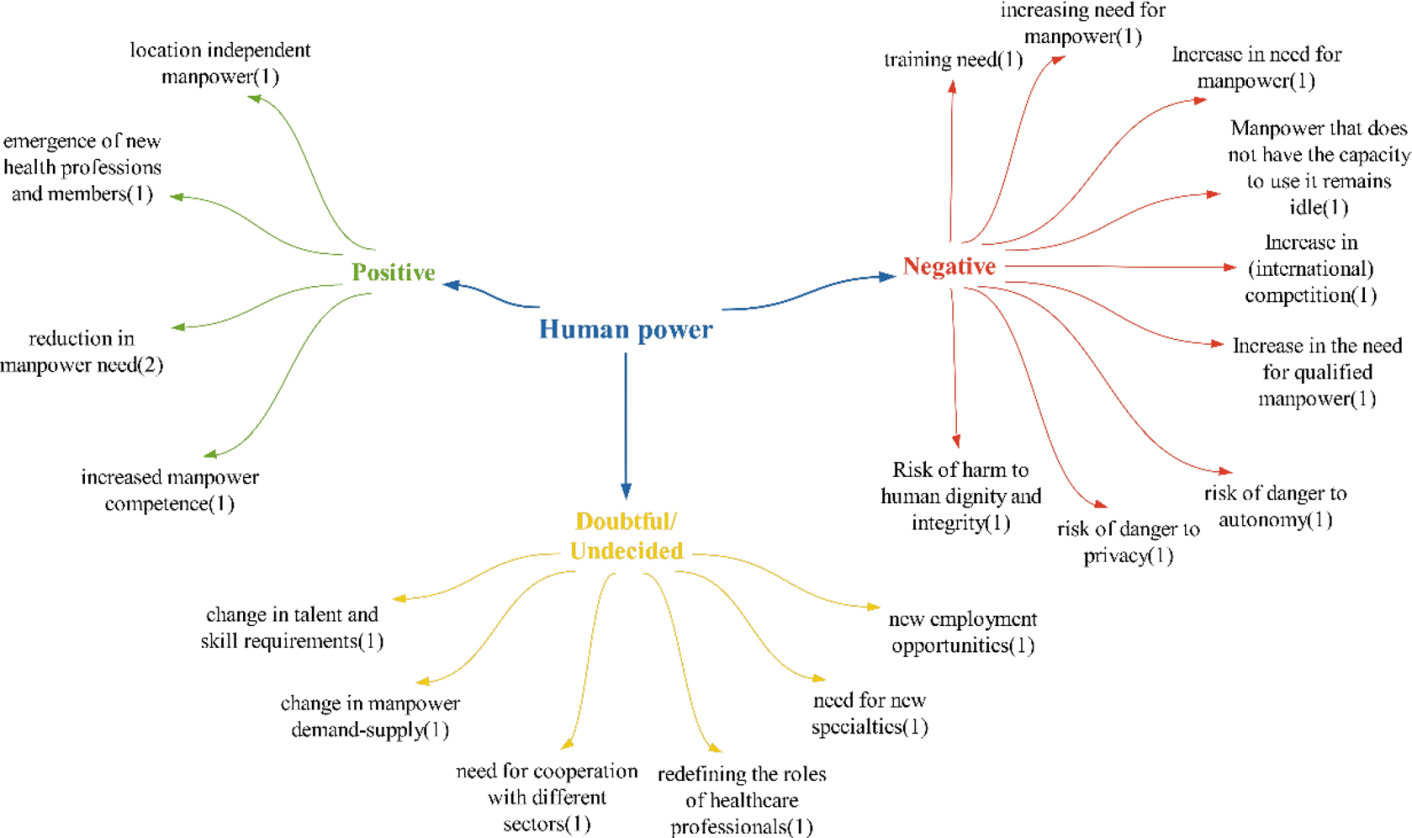
Views on what effects technologies will have on health manpower

Within the scope of the positive effects identified in the theme of manpower, it is anticipated that the “location-independent manpower effect” will become possible with technologies that virtually connect patients and healthcare workers. This is expected to make healthcare service provision independent of location, as mentioned in the literature [41]. The impact of increasing manpower competence is similar to the findings of Sarıkoç’s study, while the effect of reducing the need for manpower aligns with the research of Akalın and Veranyurt [57, 58] (Fig. 10).

Within the scope of the negative effects identified in the manpower theme, the importance of health manpower’s skills in using technologies has been highlighted in the literature, as seen in the studies of Tatlı et al. and Işık and Akbolat [59, 60]. The impact of increasing the need for education aligns with findings from the studies of Sağlam and Ünsalan and Söyler and Averberk. Similarly, the effect of increasing the need for manpower is consistent with the study by Yılmaz et al. The effect of increasing the need for manpower’s qualifications is also supported by the study of Yılmaz et al. [11, 23, 61]. The concept of the risk of harm to human dignity and integrity, danger of privacy and risk of danger to autonomy – these issues revolve around whether the use of technologies is necessary, as it directly impacts human health, and personal values, potentially endangering autonomy. The increasing need for manpower poses a dilemma, as different participants have varying opinions on whether the demand for manpower will decrease as a positive effect. The “increased effect of international manpower competition” is expected to arise in the realm of health manpower involving those proficient in using technologies, those capable of producing these technologies and individuals required for tasks such as maintenance and repair.

**Fig. 11:**
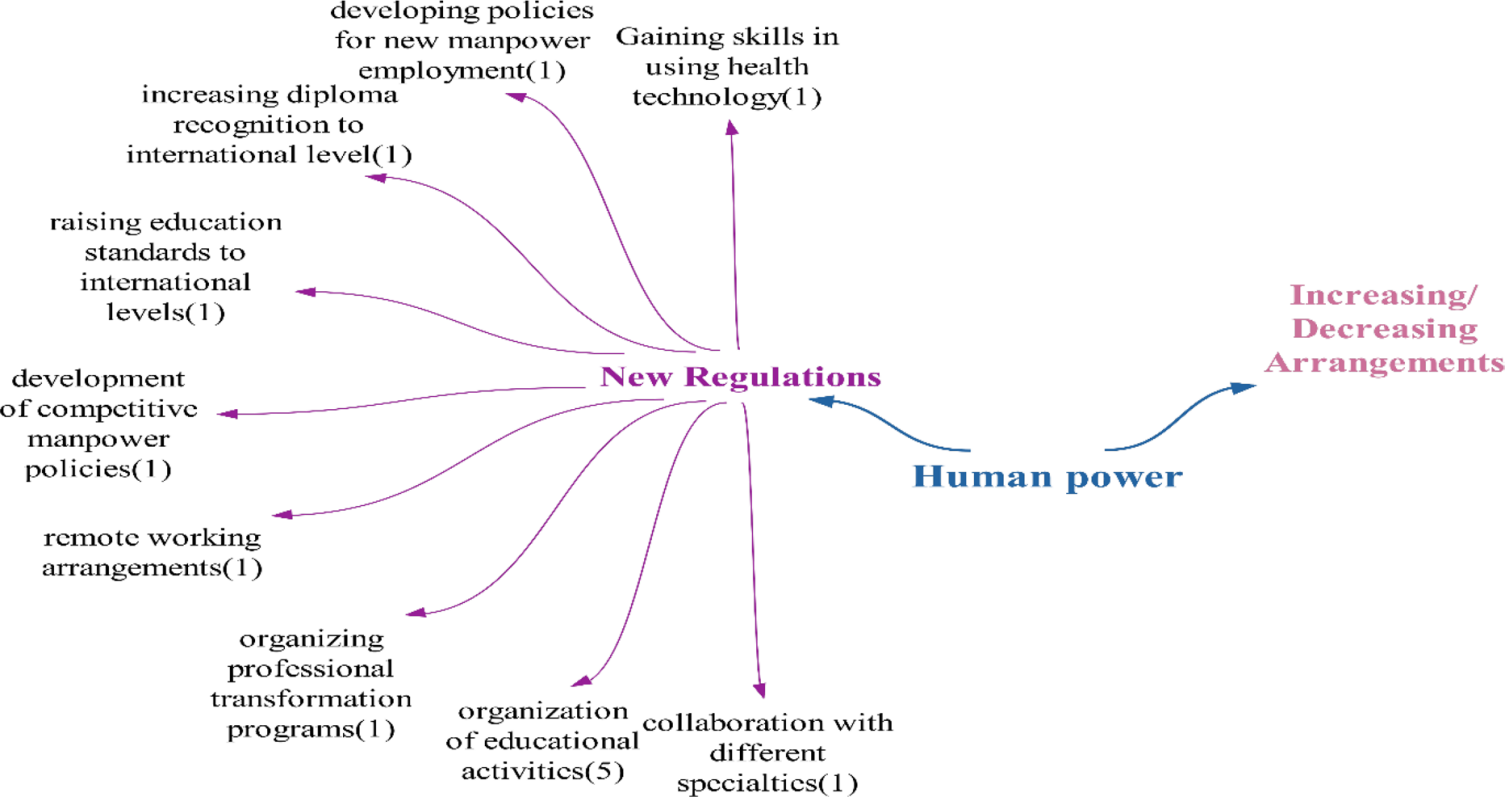
Policy recommendations regarding the impact of technologies on healthcare workforce

Considering the effects identified in the doubtful/undecided statements category, it is anticipated that new technologies will necessitate new abilities and skills, distinct from those currently utilized by healthcare personnel. These technologies represent cutting-edge engineering marvels. Given that the primary focus of healthcare personnel is health, the introduction of these technologies in healthcare may lead to diverse manpower requirements, collaboration with various sectors, the emergence of new areas of expertise and, subsequently, new job opportunities. In their analysis of the metaverse, Yılmaz et al. emphasize the need for extensive training for the health workforce due to the high level of expertise required. Since it may be challenging to consolidate various fields within in a single individual, a multidisciplinary team approach will be essential [11]. Furthermore, involvement in healthcare delivery across different sectors could prompt a re-evaluation of the roles of healthcare personnel. On the basis of participant feedback, it was concluded that technologies are likely to predominantly have a negative impact on the health workforce (Fig. 11).

Participants only made suggestions within the scope of new regulations for health manpower mainly focusing on organizing training activities for health manpower related to these technologies. One notable suggestion is implementing remote working arrangements. As future health technologies will decrease the necessity for patients to physically visit a health institution by connecting them with health providers in a digital environment, the idea of eliminating the need for health workforce to be present at the institution becomes crucial, making remote working arrangements an important suggestion (Fig. 15).

### Ethics/equity theme

**Fig. 12:**
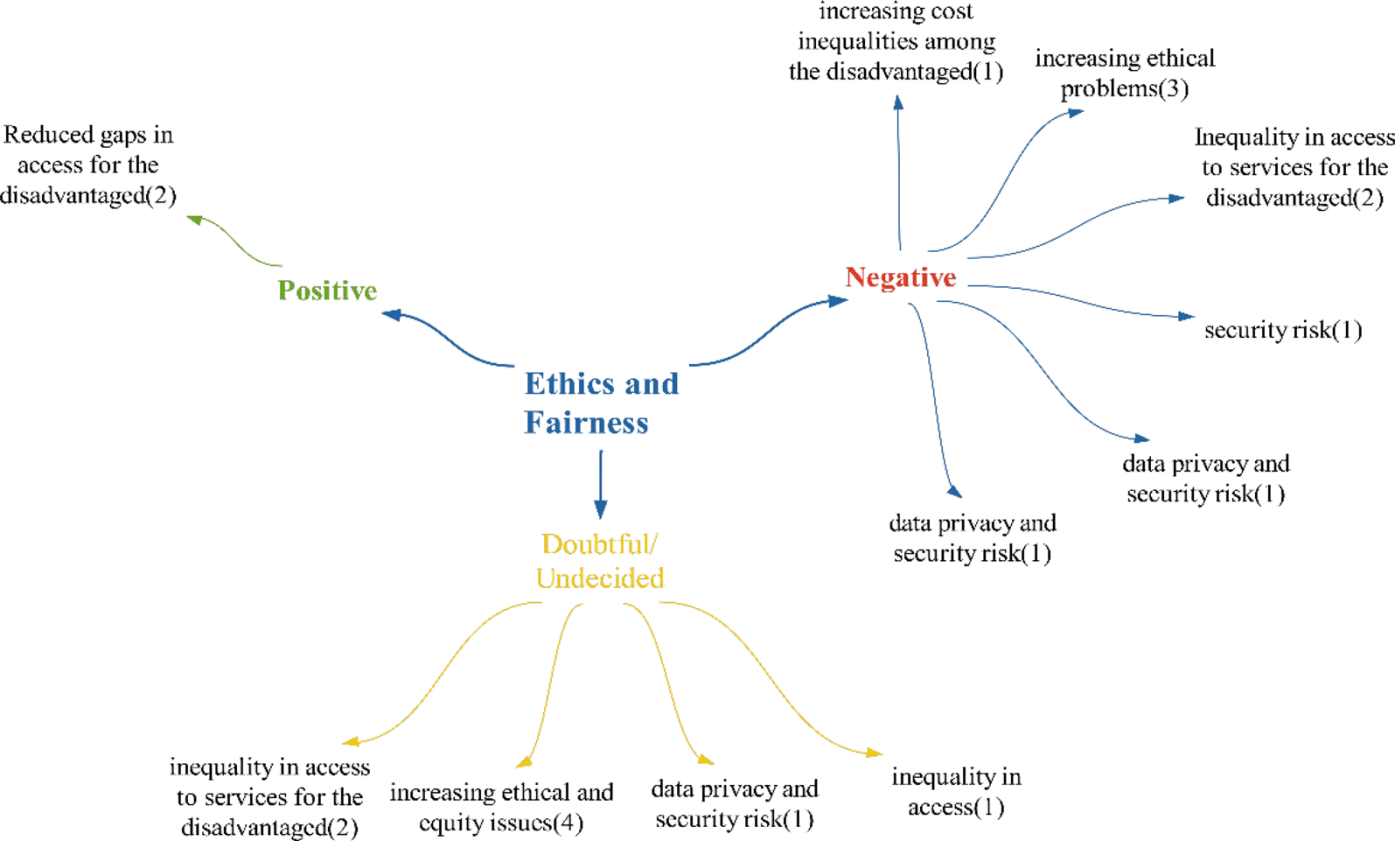
Views on what kind of effects technologies will have on ethics and equity

Participants stated that technologies will have a positive impact on ethics/fairness. Accordingly, inequalities in access to health services for the disadvantaged will decrease. However, there are also participants who state that technologies will increase inequalities within the scope of negative effects. When looking at the literature, it is controversial whether technologies will increase (e.g. [30]) or decrease [62] inequalities, as in this study (Fig. 12).

Within the scope of negative effects in the ethical fairness theme, participants mentioned security risks, an increase in ethical problems and data privacy effects. It is seen that the participants also mentioned these effects under the theme of “regulation/legislation”. The effect of disadvantaged people experiencing inequality is similar to the statements in the studies of Söyler and Averbek in the literature [23]. The effect of non-compliance with personal values, as in the “scope of regulation/legislation”, is related to whether these technologies are compatible with privacy because they directly affect human health, whether they are compatible with individuals’ values, and whether there is any coercion in their use.

**Fig. 13:**
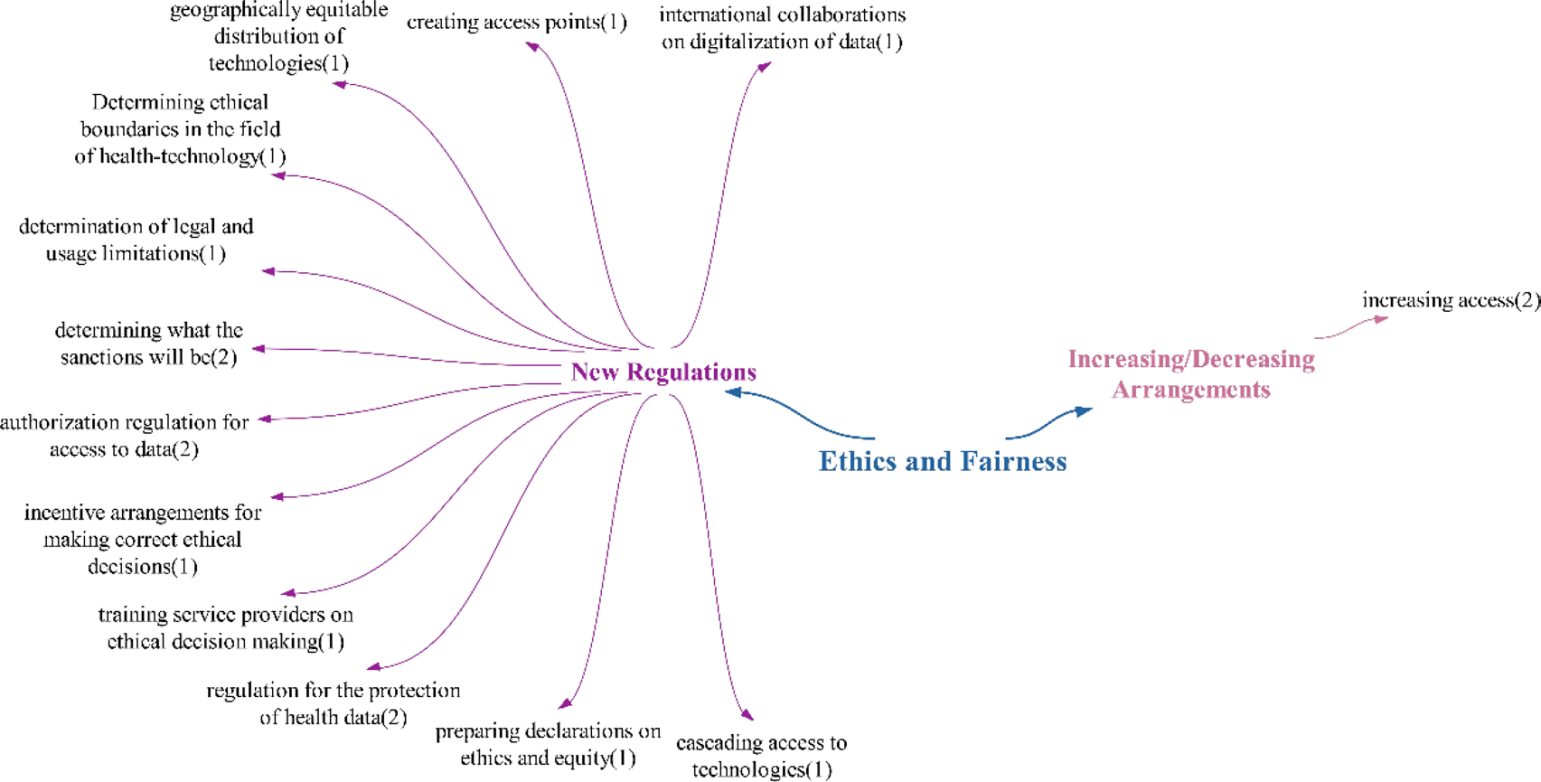
Policy recommendations regarding the ethical and equity impact of technologies

Within the scope of doubtful/undecided effects, the participants did not mention any points different from the points they mentioned in positive–negative effects. On the basis of the participants’ statements, it was concluded that technologies will mainly negatively affect ethics and equity (Fig. 13).

Participants’ policy recommendations regarding ethics and equity mainly focused on new regulations. These new regulations address issues such as the “protection of health data, authorization regulations for accessing data, and evaluating of the sanctions in cases of ethical violations”.

### Cybersecurity and biosecurity theme

**Fig. 14:**
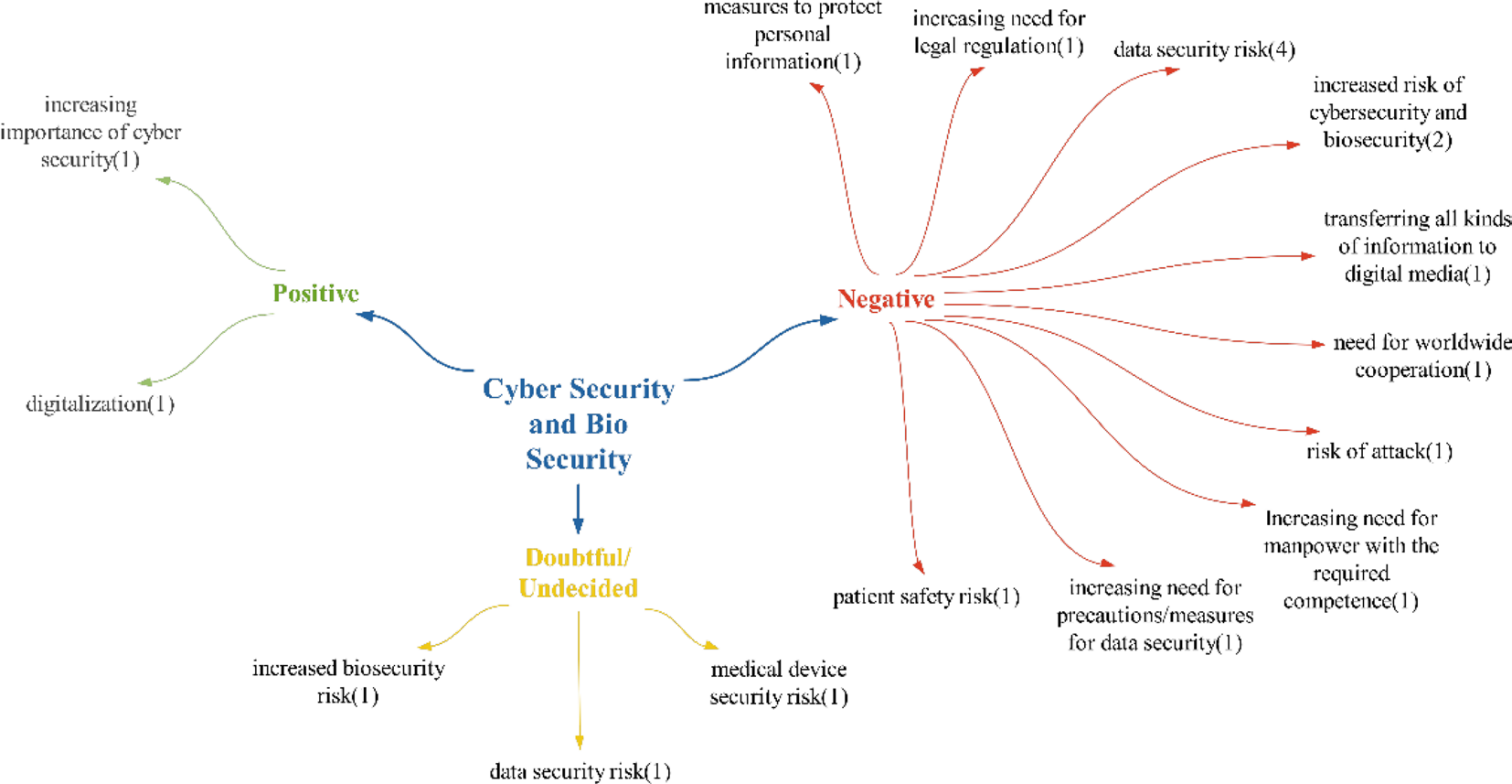
Opinions on what kind of effects technologies will have on cybersecurity and biosecurity

Within the realm of positive effects identified in the realm of cybersecurity and biosecurity, it is believed that the advancements and actions taken against cybersecurity risks will increase the importance of cybersecurity. While some participants viewed the digitization of health processes negatively, others saw it in a positive light. Therefore, it can be said that this situation is not entirely clear and is open to debate (Fig. 14).

In terms of negative effects on cybersecurity and biosecurity, the impact of data security risks align with the findings in the study by Çakır et al. [63]. As a results, it is expected that precautions/measures for data security will be necessary. Participant statements regarding personal information echo those found in studies by Dülger and Çakır et al. [63, 64]. This indicates that the need for precautions to safeguard personal information as expressed by participants is an anticipated outcome. Since the risk to data arises from the transfer of all data to the digital realm, all types of information shared by participants fall within the realm of negative effects due to this transfer. Additionally, as these risks pertain to patient data, they can be seen as contributing to the risk of cyberattack risk effects, Tosun et al. suggest in their study that the healthcare sector faces more cyber risks compared with other sectors [65]. The need for international cooperation suggests that security mechanisms developed through such collaboration will be more comprehensive and secure than those developed domestically. It is believed that training skilled personnel individuals in these areas will have a positive impact in terms of having competent manpower.

**Fig. 15:**
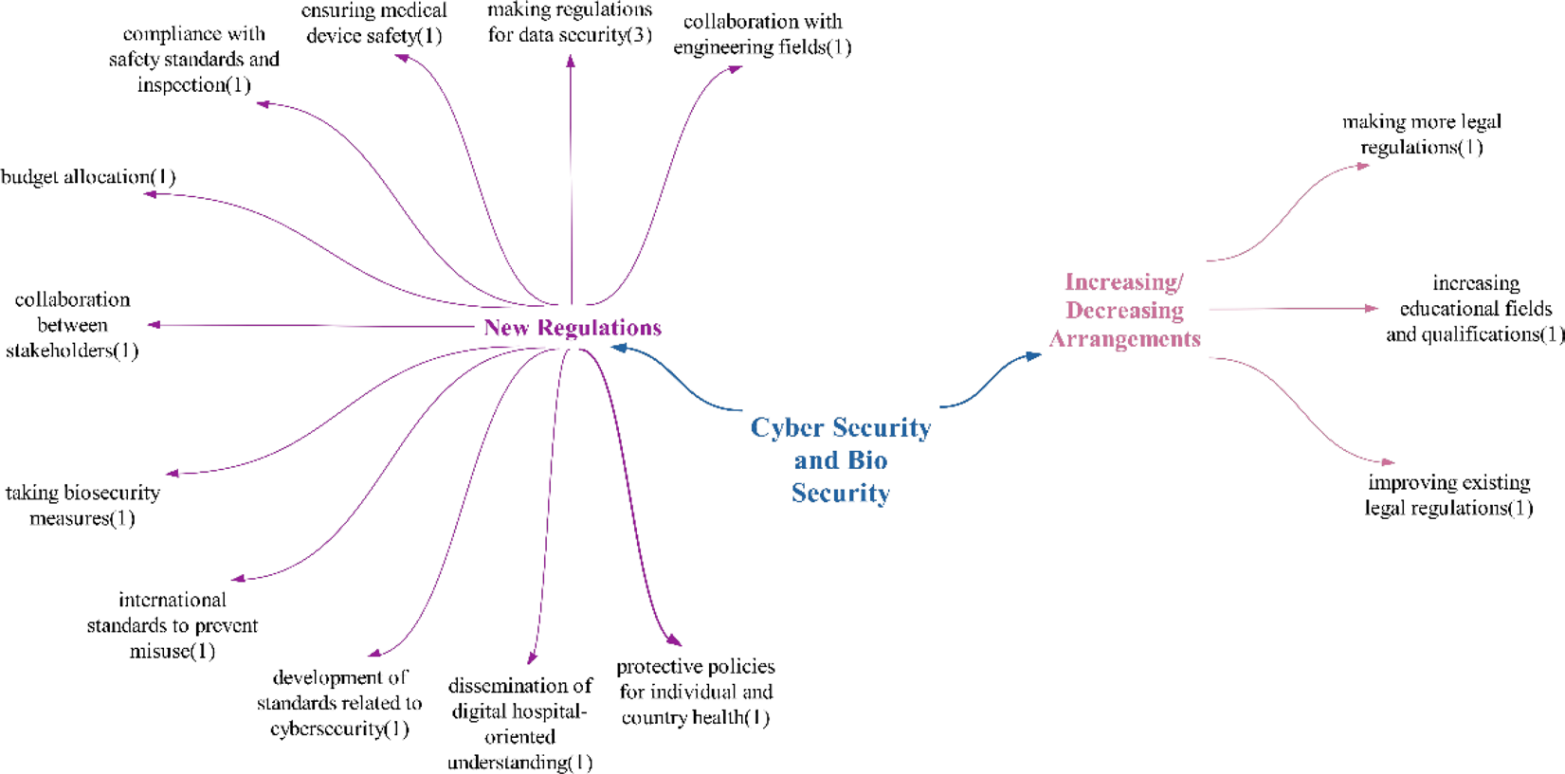
Policy recommendations on the impact of technologies on cybersecurity and biosecurity

In the doubtful/undecided statements, the participants only mentioned the medical device security risk effect, unlike in the positive–negative statements. The concerns raised include malicious attacks and the exploitation of software vulnerabilities that may arise when numerous medical devices are connected to networks. On the basis of the participants’ statements, it was determined that these technologies primarily have a negative impact of cybersecurity and biosecurity (Fig. 15).

Participants’ policy recommendations regarding cybersecurity and biosecurity mainly focused on the implementation of new regulations. Within this theme, the issue of creating regulations for data security was consistently mentioned. This is considered a crucial step, given the increasing use of technology to transfer health data into digital formats, where such data are considered both personal and health-related.

### Local, domestic and national production theme

**Fig. 16:**
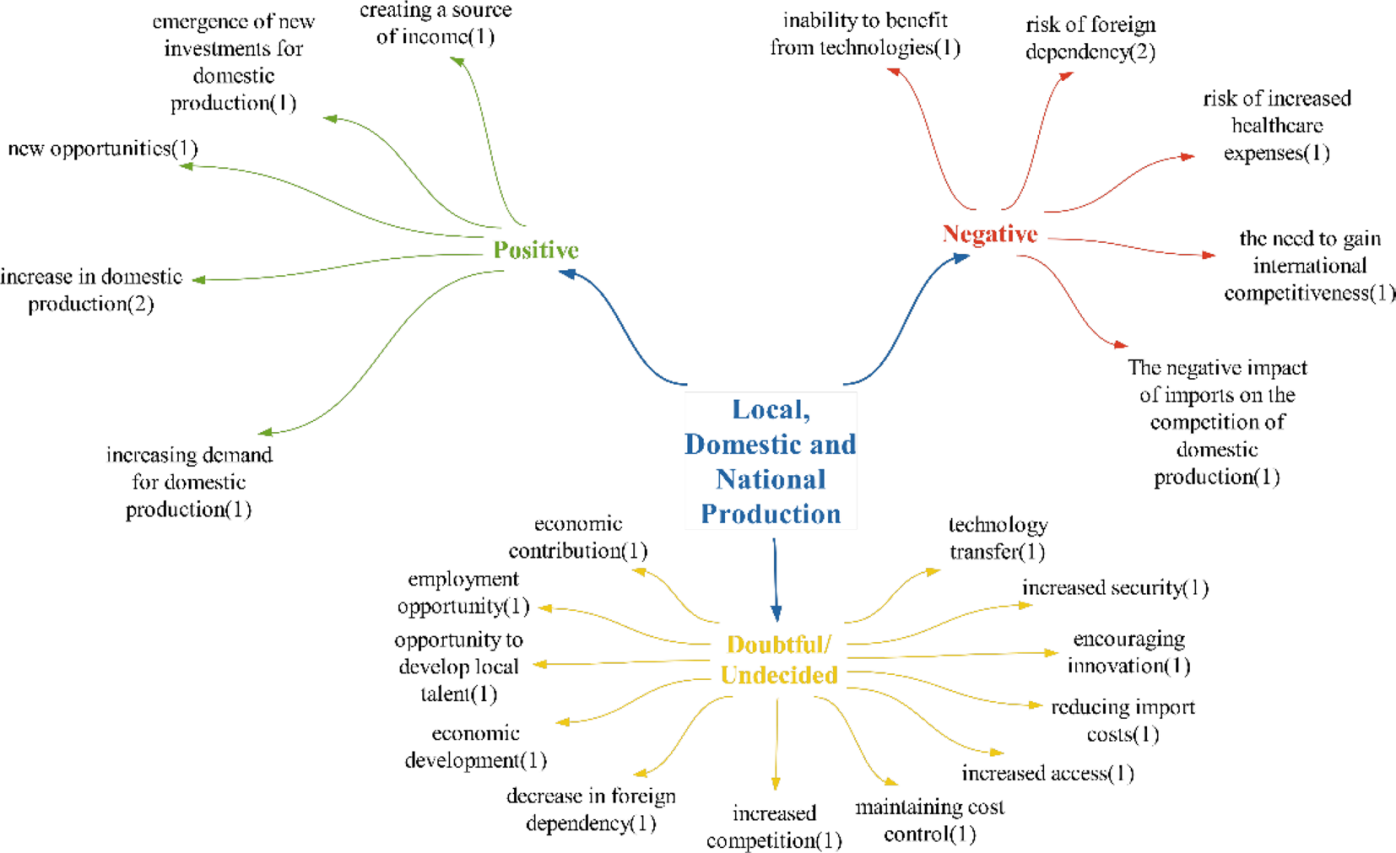
Opinions on what kind of effects technologies will have on local, domestic and national production

Within the realm of positive effects on the theme of domestic production, it is believed that Türkiye should increase its domestic production capacity without depending on foreign investments. This is because increasing domestic production can provide significant advantages to countries with technological capabilities during epidemic periods [66]. It can be noted that Türkiye is making progress in this direction, dictated by the increased desire for domestic production and investments in domestic and national production. Examples of this progress include the establishment of the Health Industries Transformation and Research Platform (SEDAP) and the publication of the Smart Life and Health Products and Technologies Roadmap. The expected result of domestic production creating a source of income will be achieved as long as domestic production continues to increase. Additionally, the emergence of new opportunities is anticipated, given that Türkiye has not yet reached a sufficient level of technological saturation, leaving many opportunities to be explored in this field (Fig. 16).

If Türkiye is lacking in the domestic production of technologies, the expected consequences include a lack of competitive power due to reliance on foreign technologies and insufficient development of domestic production. This will lead to increased health expenditures, making it difficult for many citizens to afford necessary technologies. As a result, patients’ needs may not be met, and the healthcare system will struggle to fulfil its basic functions.

Regarding whether domestic production of these technologies can be achieved in Türkiye, dubious/undecided statements were made: “More reliable devices will be a significant source of income, economic development, new employment opportunities for the workforce that will be involved in this sector, reduced foreign dependency, and increased access for the country’s citizens to these services as costs be lower compared to imports.” The effects of “increased productivity and cost control” are likely to be seen. Furthermore, it transfer through the trading of these products, and heightened competition will stimulate innovation among producers.

**Fig. 17:**
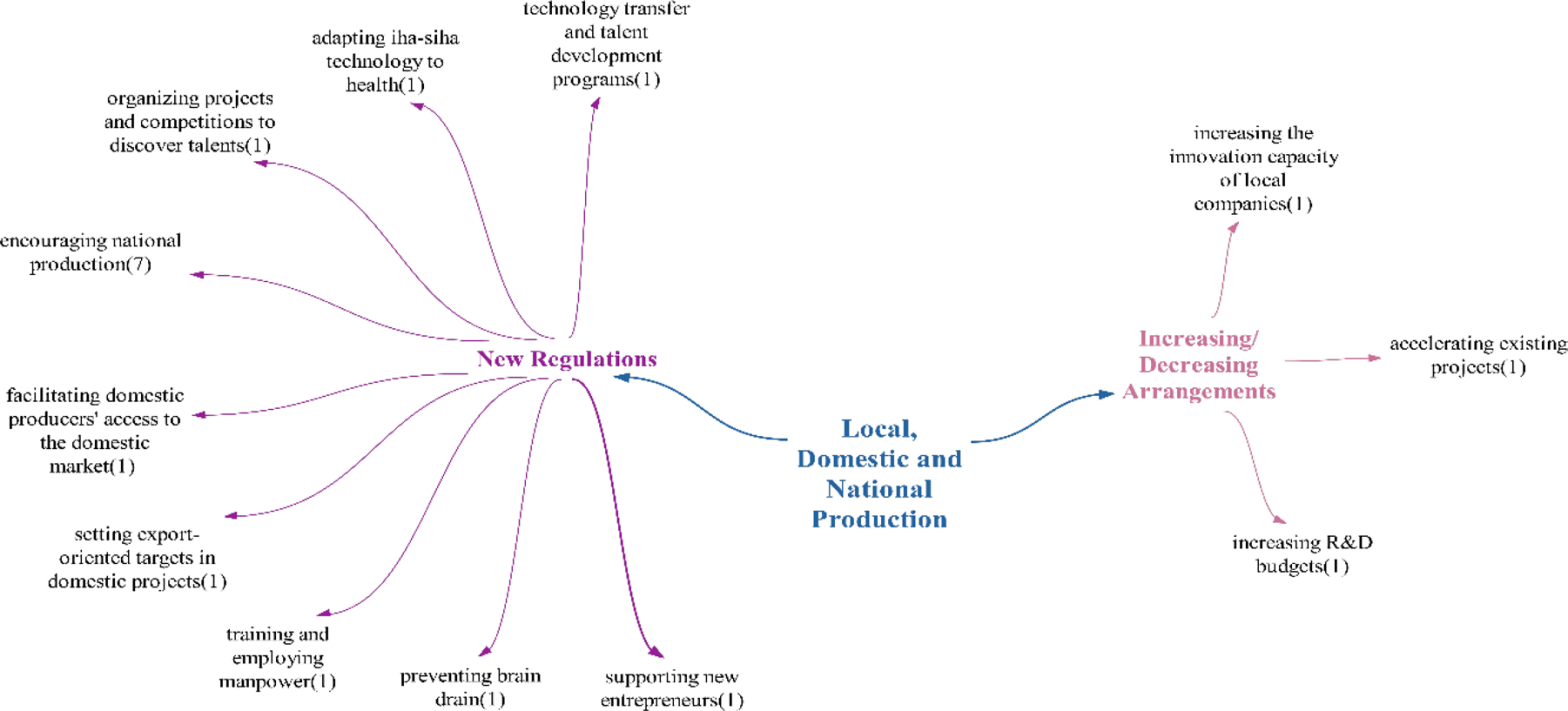
Policy recommendations regarding the impact of technologies on local, indigenous and national production

It was observed that the participants mostly made doubtful/undecided statements regarding the impact of technologies on local, domestic and national production in Türkiye. The number of statements regarding positive and negative effects is equal (Fig. 17).

Policy recommendations regarding the impacts of technology on local, indigenous and national production have primarily focused on implementing new regulations. Participants have predominantly proposed the promotion of national production. One noteworthy suggestion within this context is the prevention of brain drain. It is believed that substantial incentives should be offered to individuals capable of engaging in domestic health technology production to encourage them to remain in the country. Another notable recommendation is the utilization of “unmanned aerial vehicle/UAV(insansız hava aracı/İHA)-armed unmanned aerial vehicles/AUAV(silahlı insansız hava araçları/SİHA)” technology adapted for healthcare purposes. Given Türkiye’s achievements in the unmanned aerial vehicle (UAV) sector, it is anticipated that leveraging and applying these technologies in the healthcare industry will yield substantial advantages.

### Digital literacy theme

**Fig. 18:**
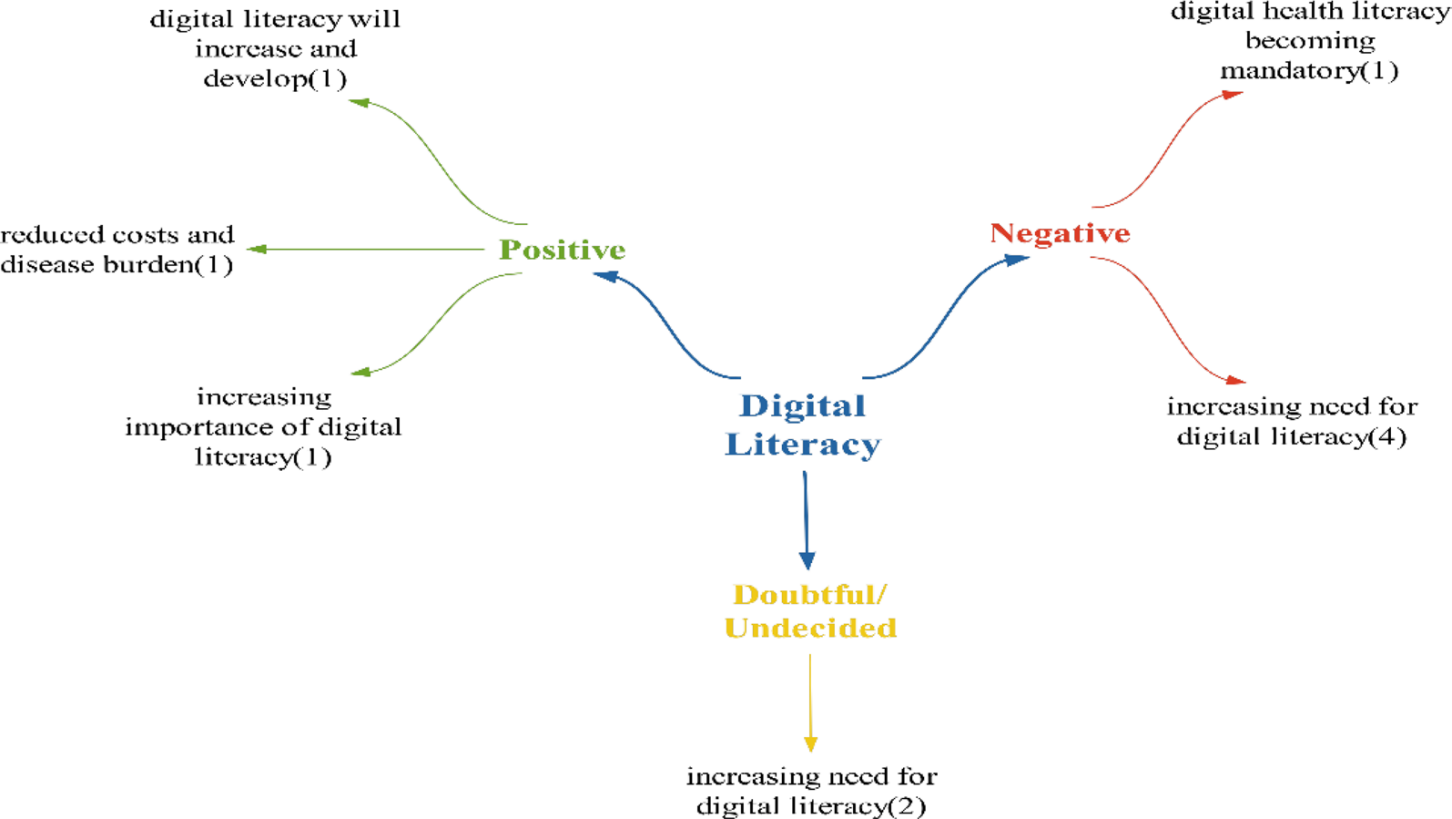
Opinions on what kind of effects technologies will have on digital literacy

Within the positive effects identified in the digital literacy theme, it is believed that the growing importance of digital literacy will help individuals recognize its significance. This is because they will need to manage their health processes in the digital environment within the future health ecosystem. It is anticipated that individuals will enhance their ability to comprehend and utilize information in the digital realm. By utilizing this information, they can safeguard their health and prevent diseases before they manifest, ultimately reducing the costs and burden associated with diseases (Fig. 18).

Regarding the negative effects on digital literacy, participants primarily highlighted the necessity for digital literacy. They emphasized the importance of individuals possessing digital skills to access and comprehend health-related information available online [67]. As healthcare processes become increasingly digitized, users are taking on more responsibilities in utilizing online information and mobile applications. Consequently, an increased demand for digital literacy is expected. The correct utilization of this information by users will impact both individual health outcomes and the quality of health services [68]. The eventual requirement for digital health literacy may become mandatory as health processes continue to undergo digital transformation, potentially eliminating paper-based transactions entirely.

**Fig. 19:**
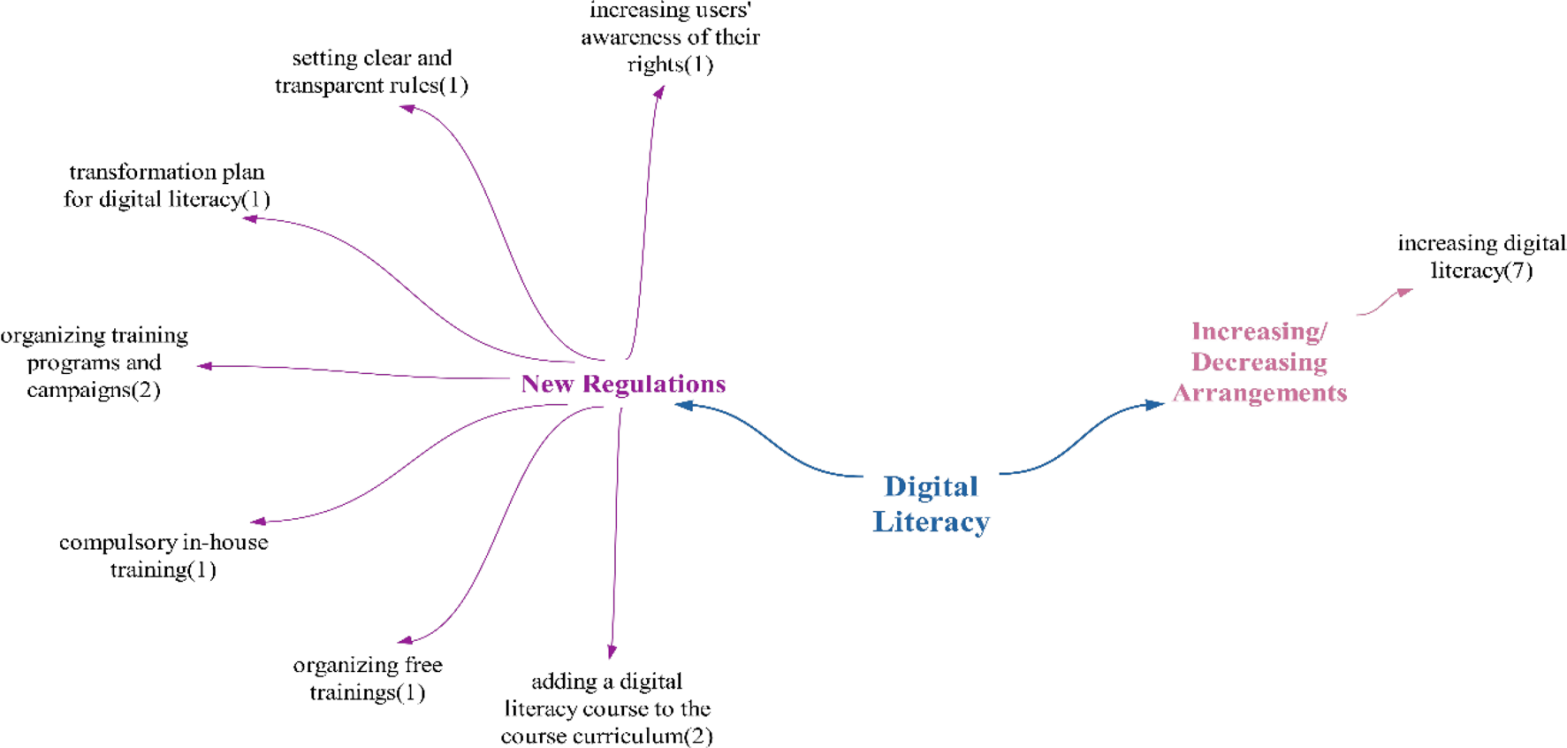
Policy recommendations regarding the impact of technologies on digital literacy

In statements categorized as doubtful/undecided, it was noted that the need for digital literacy will only escalate. Upon reviewing participant statements, it was deduced that health technologies are likely to have a predominantly negative impact on digital literacy (Fig. 19).

Although policy recommendations for digital literacy were primarily focused on new regulations, every participant also recommended increasing digital literacy. Suggestions within the realm of new regulations included adding a digital literacy course to the curriculum and implementing training programs and campaigns.

### The theme of increasing and changing needs, wants, demands and expectations

**Fig. 20:**
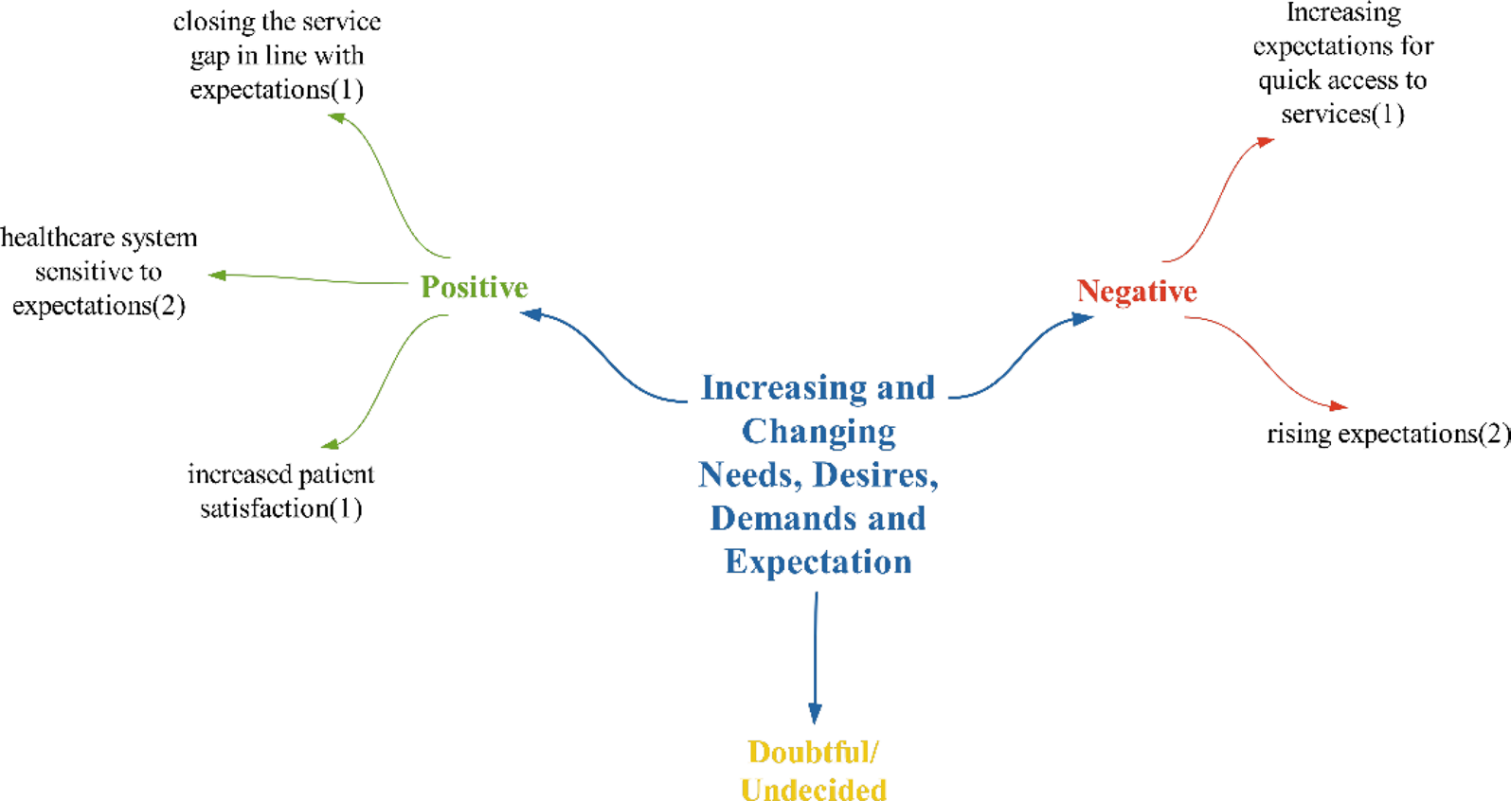
Views on what kind of effects technologies will have on increasing and changing needs, wants and demands

Within the realm of positive effects, the health system’s sensitivity to expectations is akin to the findings in the study by Yılmaz et al. In their research, Yılmaz et al. noted that solutions to expectations are beginning to emerge through the use of these technologies. [11]. It has been observed that, with the digitalization of health, there is a multifaceted communication and interaction taking place [69]. Consequently, it is anticipated that patient expectations, needs, and demands can be more accurately determined, leading to a healthcare system that is more attuned to expectations. This, in turn, may help bridge the service gap that could arise against expectations, ultimately resulting in increased patient satisfaction, which is another positive outcome. Studies have also indicated that the extent to which patients’ expectations are met influences their level of satisfaction [70] (Fig. 20).

Considering the negative effects, the rapid spread of the internet has caused individuals to spend most of their time online, whether on the internet or social media. Thoughts and ideas can be openly expressed on social media, developments can be shared with others, and people worldwide can quickly be informed about events happening anywhere [71]. In such an environment, it is expected that individuals would become more aware of health technologies and start demanding these services for themselves as they witness the benefits provided by technology. Therefore, it can be anticipated that the expectations expressed by participants will increase. It will be crucial for the health system to keep up with these expectations as individuals become more aware of developments and have higher expectations. Consequently, the necessity of managing the system in an interactive structure is another expected outcome. The impact of rising expectations for rapid access to services as expressed by participants, is another result that is expected to be experienced in line with the advancements in the new digitalized health system.

**Fig. 21:**
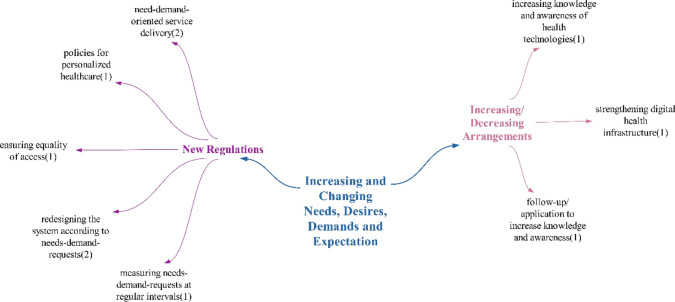
Policy recommendations regarding the impact of technologies on increasing and changing needs, desires, demands and expectations

Participants did not make any doubtful/undecided statements under this theme. Since the number of positive and negative statements used by the participants regarding the impact of technologies on increasing and changing demands and needs is equal, it has been concluded that there will not be a predominant effect (Fig. 21).

Participants primarily made suggestions regarding new regulations to address the increasing and changing needs, wishes and demands. Specifically, they discussed redesigning the system and providing services that meet expectations and requests within the scope of these new regulations.

### Costs/expenses theme

**Fig. 22:**
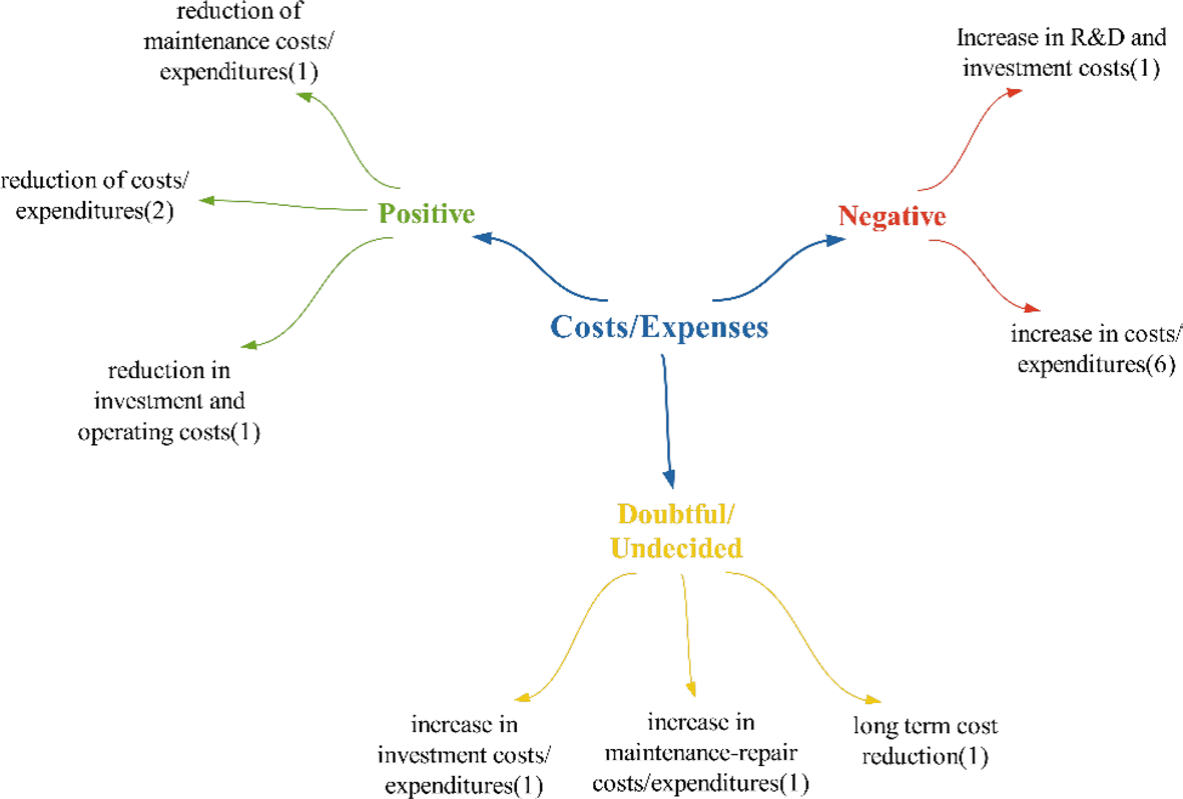
Participants’ views on what kind of impact technologies will have on costs/expenditures

As can be seen, while most participants mention that costs will increase, there are also some who believe they will decrease. Upon examining the literature, it is evident that there are conflicting statements regarding whether costs will decrease or increase, mirroring the results of the study, and indicating that the issue is not entirely clear. Çınar and Özkaya and Güven and Kılınç asserted in their studies that costs will increase. Conversely, Paksoy, Çalışkan and Çınaroğlu, Yılmaz et al. and Majumder and Deen stated in their research that technologies will reduce health costs [72–76]. Another positive effect highlighted is the decrease in maintenance costs, suggesting that higher-quality care will be provided, which aligns with the findings of Rathee et al. [77]. The final positive effect, indicating a reduction in investment and operating costs, implies that there will be no need for expenses related to physical structures, as the healthcare sector transitions to a digital environment, becoming location-independent. However, a negative effect mentioned by participants regarding costs/expenditures is the rise in research and development investment costs. Given that advanced health technologies represent a new field, they are likely to require ongoing research and development efforts, leading to increased investment costs (Fig. 22).

**Fig. 23:**
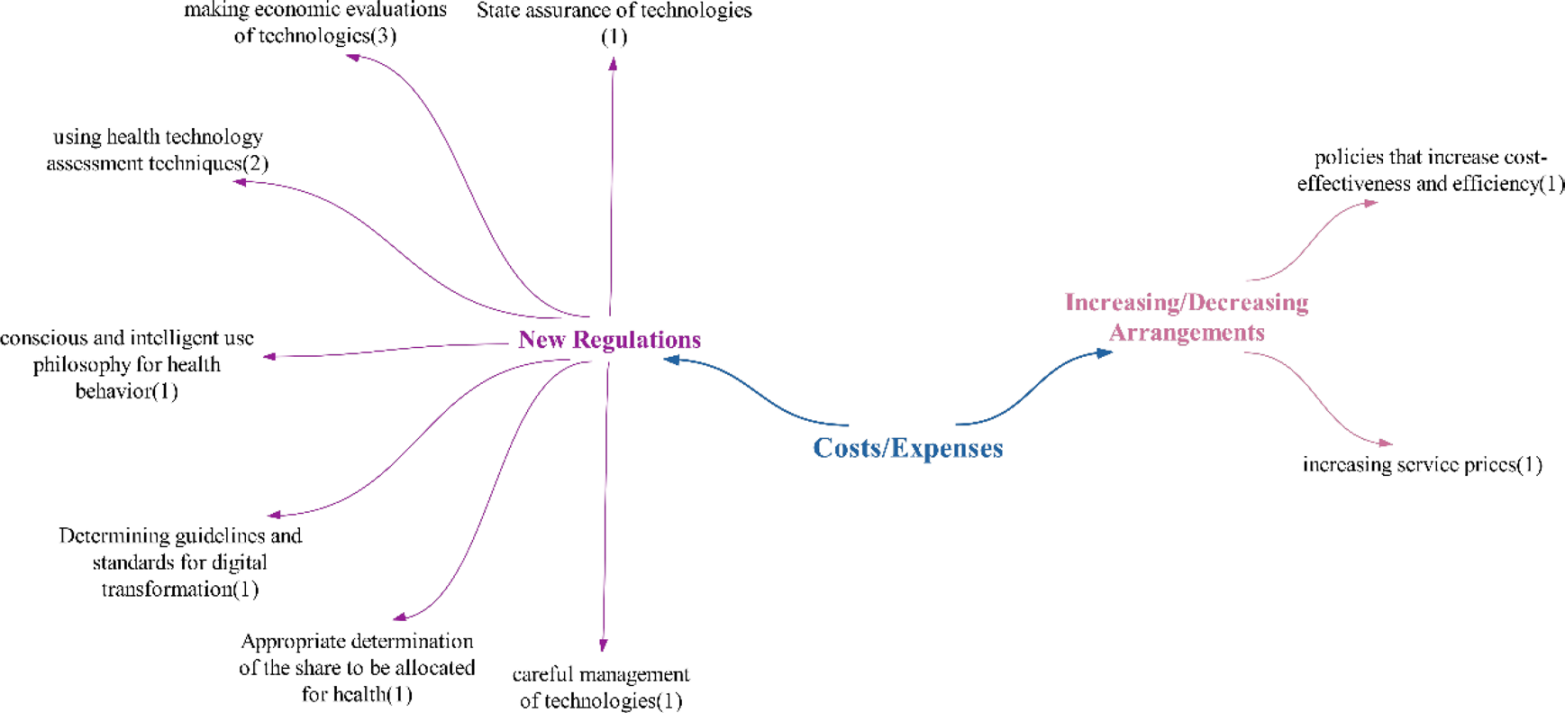
Participants’ policy recommendations regarding the impact of technologies on healthcare costs/expenditures

It has been stated that, in cases of doubtful/unstable effects, unlike positive and negative effects, cost reduction will only be achieved in the long term. In the studies of Çalışkan and Çınaroğlu, it was concluded that new technologies have the potential to improve health outcomes by providing cost savings in the long term [75]. Upon examining participant statements, it was concluded that technologies will predominantly have a negative impact on healthcare costs/expenditures (Fig. 23).

Participants primarily made suggestions within the realm of new regulations concerning healthcare costs/expenditures. The table illustrates that Türkiye will require new regulations to address the costs associated with future health technologies. The predominant suggestion in the policy recommendations provided was to conduct economic evaluations of technologies, particularly within the financing context.

**Fig. 24:**
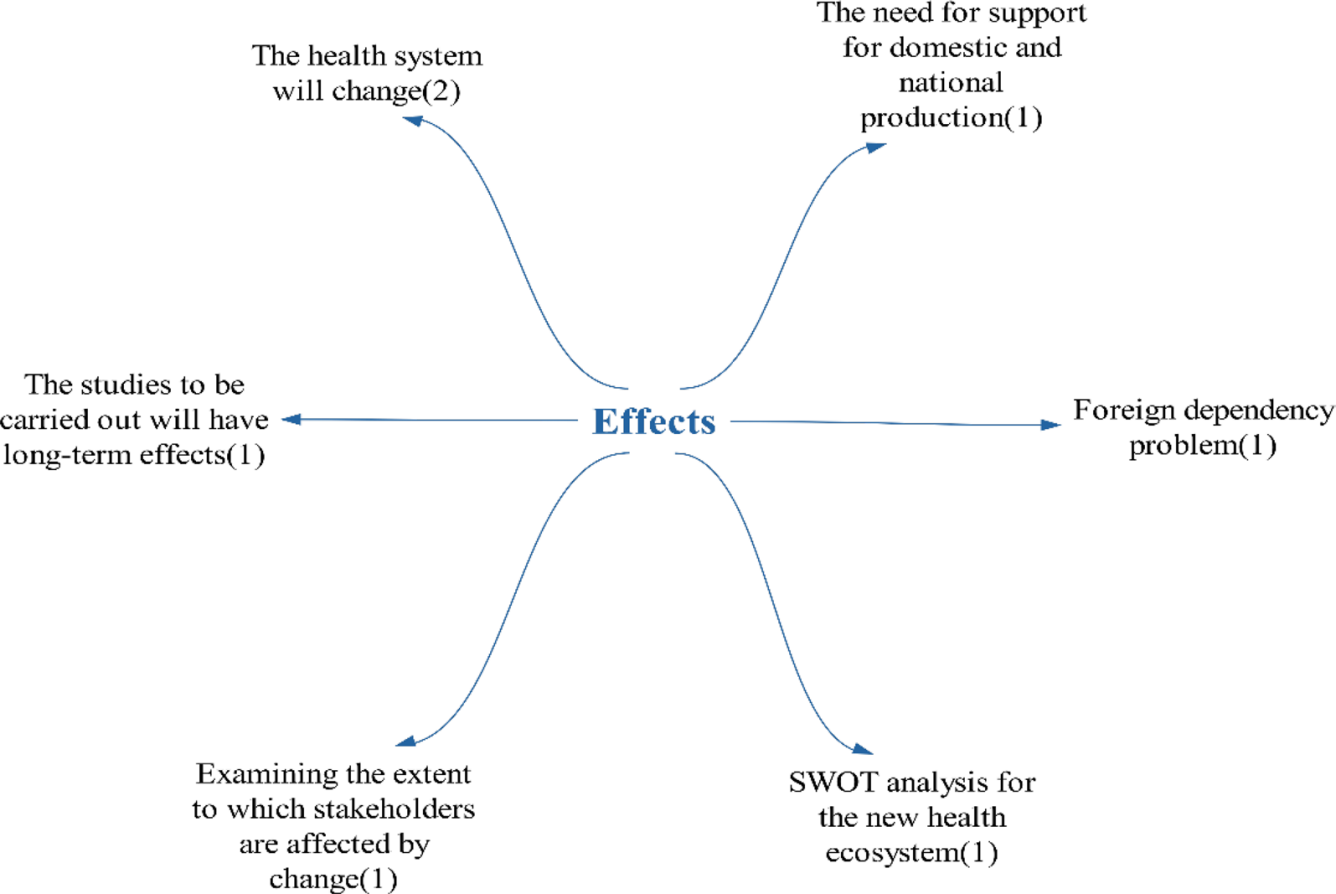
Other opinions on what kind of impact technologies will have on the healthcare ecosystem. SWOT, strengths, weaknesses, opportunities, and threats

The statements regarding the eleventh question of the interview form, “What will be the impact of technologies on the health ecosystem”, are presented in Fig. 24.

A participant commented on the last question of the interview form, stating “different points you would like to add”, and emphasized the necessity of international production for these technologies.

“It is recommended that our country’s health system be reconsidered with an approach based on producing for the world in Türkiye, without restricting it within our own borders...”

## Conclusion and recommendations

Within the scope of the study’s findings, the following suggestions are proposed to shape Türkiye’s health system in accordance with the future health technology trends that will shape the health ecosystem:

*Controversial areas:* It has been determined that technologies will have controversial effects on certain topics. These topics include access (whether access will increase), financing (whether the financial burden will increase), manpower (whether the need for manpower will increase), ethics and fairness (whether inequalities will increase) and costs/expenditures (whether costs will increase). Similarly, in the literature, it is evident that there is no consensus on these areas, with contradictory statements found in different studies. It is recommended to conduct more specific studies on these issues.

*Current follow-up on recent developments:* The study found that participants consistently discussed the importance of staying current with technology across various themes such as access, regulation/legislation, and manpower. On the basis of the findings of the study, it is recommended that institutions or teams be established in Türkiye to systematically and regularly monitor technological advancements.

*Improving digital literacy:* This was a common topic of discussion among participants, who highlighted various themes such as access, service delivery, and digital literacy. To enhance digital literacy, it is recommended to organize training programs, offer free seminars, incorporate in-service training plans and integrate them into course curricula. Additionally, educational and engaging software can be created to facilitate digital health processes for children. It is advisable to encourage both young and adult individuals to plan and monitor their health processes regularly in the digital environment to reap numerous benefits. Free training sessions can be held at public education centres, and primary care physicians can refer their patients to these sessions.

*Local production:* This was a major topic of discussion among participants, covering various themes such as financing, regulation/legislation and local, indigenous and national production. It is believed that domestic production is crucial, as it can provide opportunities such as decreasing foreign dependency, lowering import costs, ensuring safer devices, generating income and improving access to technology for citizens. Therefore, it is suggested that ongoing studies in Türkiye be expedited, incentives for new research be offered, and support entrepreneurs in this field be provided.

*Making economic evaluations of technologies:* There are various themes that are often discussed when it comes to making economic evaluations of technologies, including financing, service delivery and costs/expenditures. One notable suggestion is the creation of an independent organization in Türkiye that would be responsible for conducting economic evaluations of Technologies, similar to the National Institute for Health and Care Excellence (NICE) in the UK. Another frequently emphasized issue is the shift towards a value-based payment model.

*Improving health technology assessment (HTA) practices:* This has been a topic of discussion under various themes, such as financing and costs. Emphasizing the importance of considering the reports generated from these practices has been a key point raised multiple times.

*Making legal regulations:* The participants discussed the necessity of legal regulation for the new health ecosystem under different themes, including regulation/legislation, cybersecurity and biosecurity. Data security was highlighted as a key concern. With the digitalization of all processes, it is crucial to establish robust legal regulations to ensure there are no gaps. The digitalization of health data, which includes personal information, could make it vulnerable to malicious attacks and misuse. Moreover, it is important determine the sanctions for bad medical practices and whom they will be applied to. Identifying the licenses required to use these technologies is also essential, as health directly impacts human life.

*Collaborations:* The study discussed the issue of cooperation between the fields of health and engineering under different themes, including regulation/legislation, manpower, cybersecurity and biosecurity. This is not surprising, considering that technologies are products of engineering and are used in the field of health. It is recommended to organize multidisciplinary trainings and form multidisciplinary work teams. Collaboration with programmers who can create more user-friendly software for healthcare processes in the digital environment is also advised. By identifying major problems experienced by patients and healthcare professionals and developing solution-based applications for these issues, software developers can create valuable outputs for the healthcare field.

*Infrastructure regulations for health institutions:* These vary across different levels in Türkiye, resulting in disparities in available facilities. Consequently, not all institutions have the necessary infrastructure to provide advanced technologies. To promote equal access to these technologies nationwide, it is advisable to incentivize the development of adequate infrastructure in as many institutions as possible and implement support programs.^1^

*Ensuring equality of access:* To enhance access to the new health ecosystem created by technologies, it is recommended to develop user-friendly health applications that are compatible with voice commands. This will enable older individuals to easily navigate digital health processes. Additionally, a participant in the study suggested collaborating with internet providers. It is proposed that citizens be granted a specific amount of internet rights that can be utilized in health applications.

Our study evaluated the effects of future health technologies on the health system in terms of policy and governance, using Türkiye as an example. It is concluded that the future health technologies included in the study and their effects on health systems in terms of policy and governance can serve as a reference for studies in other countries’ health systems.

## Data Availability

Our data consist of the participants’ responses to interview questions. Due to high-level status of our participants as bureaucrats, managers and policy-makers, we are unable to share the data.

## Abbreviations

TUSAP: Türkiye Health Platform (Türkiye Sağlık Platformu)
TUBITAK: Scientific and Technological Research Council of Türkiye (Türkiye Bilimsel ve Teknik Araştırma Kurumu)
HMO: Health Maintenance Organization
NICE: National Institute for Health and Care Excellence
SEDAP: Health Industries Transformation and Research Platform (Sağlık Endüstrileri Dönüşüm ve Araştırma Platformu)

## Footnotes

1 The study originated from the master’s thesis authored by Elif Sena Kambur, which was supervised by Hasan Hüseyin Yıldırım. An abstract of the study was presented at the 6th International and 16th National Health and Hospital Management Congress in Konya, Türkiye.
